# Fast Bulky Anion Conduction Enabled by Free Shuttling Phosphonium Cations

**DOI:** 10.34133/2021/9762709

**Published:** 2021-08-31

**Authors:** Xiaolin Ge, Yubin He, Kaiyu Zhang, Xian Liang, Chengpeng Wei, Muhammad A. Shehzad, Wanjie Song, Zijuan Ge, Geng Li, Weisheng Yu, Liang Wu, Tongwen Xu

**Affiliations:** ^1^CAS Key Laboratory of Soft Matter Chemistry, Collaborative Innovation Center of Chemistry for Energy Materials, School of Chemistry and Materials Science, University of Science and Technology of China, 96 Jinzhai Road, Hefei, Anhui 230026, China; ^2^School of Chemistry and Material Engineering, Huainan Normal University, Huainan, Anhui 232001, China

## Abstract

Highly conductive anion-exchange membranes (AEMs) are desirable for applications in various energy storage and conversion technologies. However, conventional AEMs with bulky HCO_3_^−^ or Br^−^ as counterion generally exhibit low conductivity because the covalent bonding restrains the tethered cationic group's mobility and rotation. Here, we report an alternative polyrotaxane AEM with nontethered and free-shuttling phosphonium cation. As proved by temperature-dependent NMR, solid-state NMR, and molecular dynamics simulation, the phosphonium cation possesses a thermally trigged shuttling behavior, broader extension range, and greater mobility, thus accelerating the diffusion conduction of bulky anions. Owing to this striking feature, high HCO_3_^−^ conductivity of 105 mS cm^−1^ at 90°C was obtained at a relatively lower ion-exchange capacity of 1.17 mmol g^−1^. This study provides a new concept for developing highly conductive anion-exchange membranes and will catalyze the exploration of new applications for polyrotaxanes in ion conduction processes.

## 1. Introduction

Highly conductive anion exchange membranes (AEMs) are still the bottleneck of various energy generation/conversion technologies where fast anion transfer is required. Typically, AEMs are composed of a mechanically robust polymer backbone, tethered cationic groups, and free mobile counter anions like OH^−^, Br^−^, and HCO_3_^−^. Previously, high OH^−^ ion conductivity has been primarily achieved due to its faster transport kinetics with a combination of Grotthuss mechanism and diffusion mechanism [1, 2]. However, the conduction mechanism of more bulky anions like HCO_3_^−^ and Br^−^ was much less studied, and low bulky anion conductivity of AEM leads to challenges in its electrochemical applications. For example, in a fuel cell, switching the oxidant from CO_2_ free air to ambient air would lead to 50% performance loss due to the conversion of OH^−^ to HCO_3_^−^ [3–5]. In flow batteries where SO_4_^2-^ or Cl^−^ is employed as the charge carrier, AEMs usually exhibit high area resistance, resulting in lower operational current density than proton exchange membranes [6, 7].

**Figure 1:**
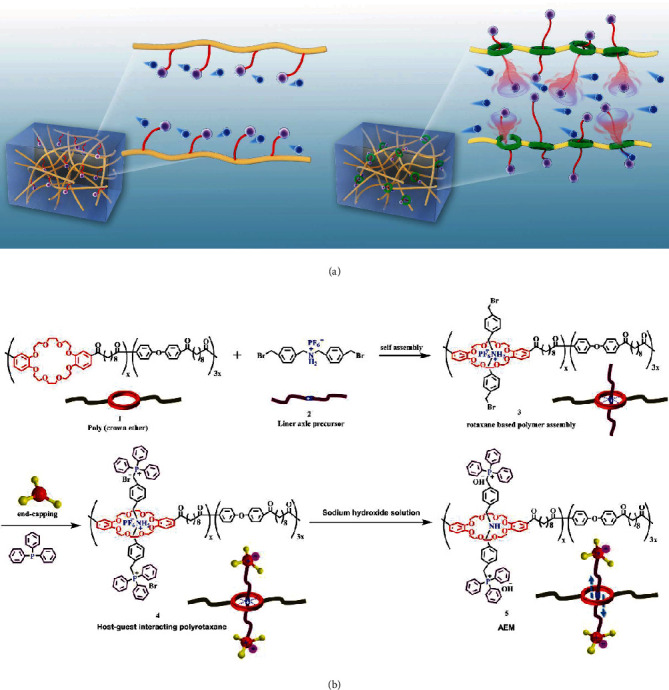
(a) Schematic illustration of polymer structure and ion conduction process in conventional tethered AEM and polyrotaxane AEM. (b) Synthetic procedure for polyrotaxane AEM from poly(crown ether) host 1 and linear guest 2.

The OH^−^ ions are considered to conduct via the Grotthuss mechanism [8–11]. As shown in Figure S17, the hydroxide ions are naturally hydrated by the hydrogen bonding with water molecules to form different hydration complexes (inactive OH−(H_2_O)_4_, active OH−(H_2_O)_3_). The continuous interconversion between hydration complexes driven by fluctuations in the solvation shell of the hydrated ions results in facile OH^−^ transport. On the other hand, the bulky anions like Br^−^ and HCO_3_^−^ can hardly form such a hydrogen bonding network with water, thus could not be conducted via the Grotthuss mechanism. Besides, bulky anions are also less mobile, less hydrated, and less dissociated from the ion-conducting groups [12]. These intrinsic disadvantages of bulky anions have (i.e., structural diffusion) [13] led to much lower ion mobility than the OH^−^ ion (relative to OH^−^ in infinite dilution H_2_O solution: Mobility_HCO3−_ = 0.23, Mobility_Cl_^−^ = 0.40) [14]. Offsettingthe low ion mobility by increasing the ion concentration often leads to excessive water swelling [15, 16]. A more realistic strategy is creating a graft architecture in which ionic graft chains are covalently bonded to the hydrophobic polymer main chains (Figure 1(a)). This architecture can form water-filled conductive channels via the self-assembly of the hydrophilic side chains [17]. Although creating such an interconnected ion conduction pathway could reduce the tortuosity during ion conduction [14], this strategy cannot resolve bulky anions' intrinsic low mobility. Consequently, the deliverable bulky anion (Br^−^, HCO_3_^−^, etc.) conductivities are generally <60 mS cm^−1^ at 80°C, which cannot meet the requirement of practical applications, e.g., >100 mS cm^−1^ for fuel cell application [14].

Pioneer molecular dynamics research revealed that solvated anions diffuse along the tethered cationic groups on the polymer backbone [18]. The transport across two cationic groups is the rate-determining step and relies on cationic groups' rotation and motion [19]. The tethered cationic group also influences the anion transport via cation-anion Coulombic interactions and cation-water dipole interaction [20]. Fast cation motion would enhance the anion mobility by contributing to the fluctuation and rearrangement of the solvation shell of the hydrated anion [21, 22]. Since both the mobility and the number of charge carriers determine conductivity [23], the dissociation ability of anions from tethered cationic groups should also be considered. Bulk anions have much lower solvation enthalpy than hydroxyl ion [24]. Tethering the cations close to each other will also decrease the entropy gain in the dissociation process [12]. Consequently, ~60% of the anions exist in the form of ion pair which are unavailable to participate in the conduction process [22]. These pioneer results suggest that the cationic group's basicity or size should be improved to facilitate anion dissociation ability and hinder ion pair formation [25]. Furthermore, the cationic group's higher mobility can contribute to enhance anion transport via enhancing the solvation shell fluctuation [21, 22] and reducing the energy barrier when transferring between two cationic groups [19].

Graft polyrotaxanes are mechanically interlocked macromolecular architectures which contains free shuttling guests encircled by macrocyclic hosts (e.g., crown ethers) [26] and provides a new direction for enhancing ion conduction kinetics. In our previous studies, we have demonstrated that the translational shuttling of ion conducting group in a polyrotaxane membrane can promote the Grotthuss conduction of H^+^ and OH^−^ by facilitating the break and regeneration of hydrogen-bonded network [27, 28], enabling high H^+^ conductivity of 260.2 mS cm^−1^ or OH^−^ conductivity of 122 mS cm^−1^. This study finds that the stimuli trigger motion and rotation in a molecular machine can also promote the diffusion conduction mechanism to deliver high bulky anion conductivity. Furthermore, the shuttling behavior was experimentally demonstrated by temperature-dependent NMR and SSNMR characterization, and the interrelationship between shuttling behavior and ion conductivity was interpreted by DSC and molecular dynamic simulation.

## 2. Results and Discussion

As shown in Figure 1(b), we have assembled a polyrotaxane AEM which contains a noncovalently tethered and free shuttling cationic group. In this mechanically interlocked macromolecular architecture, a linear molecular guest was firstly mechanically confined within a crown ether macrocyclic host, then end capped with two steric hindrance phosphonium cations. NOESY 2D NMR well elucidated the host-guest intermolecular interaction. Unlike tethered quaternary ammonium group in conventional AEMs, the noncovalently tethered phosphonium cations have exceptionally higher mobility and broader motion range as evidenced by molecular dynamics simulation. Solid-state and temperature-dependent NMR also revealed more obvious free shuttling behavior in response to increasing temperature. Although the favorable advantages originated from the polyrotaxane architecture, the phosphonium cation possesses a high pK_b_ value [29] to improve anion dissociation and larger bulky size to prevent the ions from the formation of condensed ion pairs. Consequently, the as-designed AEM showed high Br^−^ conductivity of 112.1 mS cm^−1^ and HCO_3_^−^ conductivity of 105 mS cm^−1^ at 90°C.

As an upgrade to the traditional AEM with covalently tethered cationic groups, the polyrotaxanes AEM with free shuttling phosphonium cations was constructed by threading a linear guest into the cave of a poly(crown ether) (Figure 1(b)). The linear guest has two benzyl bromide end groups, which were then end-capped with two bulky triphenylphosphine moieties. As depicted in Scheme S1, the poly(crown ether) host 1, which was synthesized via polyacylation, possesses abundant electro-dominating oxygen groups, can selectively recognize the electron-deficient sec-ammonium groups by forming multiple hydrogen bonding [30]. Therefore, a sec-ammonium containing linear guest 2 was synthesized by multistep reactions (Scheme S2-4). Firstly, the condensation reaction between amine and aldehyde group yielded a Schiff base (-C=N-C-) precursor. Treatment with lithium aluminum hydride (LiAlH_4_) can simultaneously reduce the Schiff base to secondary amine and the terminal ester group to hydroxyl groups. Lastly, the terminal hydroxyl and sec-amine groups were converted into bromomethyl and sec-ammonium groups via a sequential reaction with hydrobromic acid and ammonium hexafluorophosphate (NH_4_PF_6_). The obtained linear guest 2 was then threaded into poly(crown ether) host 1 by string in nonpolar solvent for 24 hours. The axle termini were end-capped with two bulky triphenylphosphines to introduce free shutting phosphonium cations as ion conducting groups 4. The bulky nature of phosphonium cations can constrain the liner guest within the caves of crown ether to prevent diffusional loss of the side chains [31].

**Figure 2:**
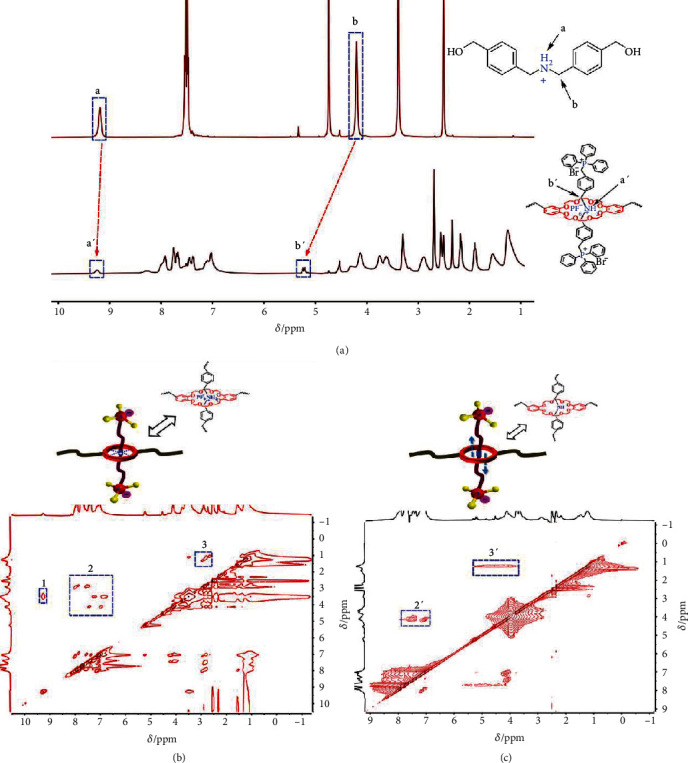
(a) ^1^H NMR of the linear guest 1, hydrogen-bonded polyrotaxane 4 (400 MHz, DMSO-*d6*, 298 K). (b) NOESY 2D NMR of hydrogen-bonded polyrotaxane 4 (400 MHz, DMSO-*d6*, 298 K). (c) NOESY 2D NMR of free shuttling polyrotaxane 5 (400 MHz, DMSO-*d6*, 298 K).

The successful assemble of hydrogen bond polyrotaxane 4 was firstly confirmed by ^1^H NMR spectra. As depicted in Figure 2(a), after threading the linear guest 2 into poly crown ether host 1, the NMR signal of sec-ammonium proton down-shifted from *δ* = 9.15 ppm to 9.25 ppm, and the methylene b proton down-shifted from *δ* = 4.19 ppm to 5.22 ppm because of the electro-donating effect of multiple oxygen groups in crown ether. Besides, the nuclear Overhauser effect spectroscopy (NOESY) was conducted to further reveal the correlation between the macrohost and the linear host (Figure 2(b)). The correlation signal in region 1 is originated from the intermolecular electron-transfer between -CH_2_- moieties in crown ether and the sec-ammonium moiety. The signal in region 2 reflects the interaction between -CH_2_- and phenyl rings in the linear guest. This result indicated that the linear side chain as well as terminal phosphonium cations was encircled by the macrocyclic hosts, i.e., crown ether, owing to the strong hydrogen-bonding interactions between the components.

The host-guest interaction is realized by the hydrogen bonding between crown ether and –NH_2_^+^-group. NaOH treatment could convert the –NH_2_^+^- group into –NH- group, thus eliminating the hydrogen-bonding interactions to free the linear host and yield free shuttling polyrotaxane 5. As shown in its ^1^H NMR spectrum (Figure S8), after converting the sec-ammonium to sec-amine, the proton signal at *δ* = 9.25 disappeared, and a new proton signal at *δ* = 5.31 ppm appeared. Meanwhile, compared with the NOSEY NMR spectrum of hydrogen-bonded polyrotaxane 4 (Figure 2(b)), the NOESY NMR of free shuttling polyrotaxane 5 (Figure 2(c)) did not show any signal for -NH_2_^+^- and -O-CH_2_-CH_2_-O- intermolecular interaction in region 1. This result confirmed no covalent bonding or noncovalent bonding between the linear guest and poly (crown ether) host in free shuttling polyrotaxane 5. The resulting high mobility and extended motion range of phosphonium cation are expected to effectively promote the diffusion conduction of anions. To rule out the possibility of intramolecular H-H coupling, a COSY experiments had been conducted. As shown in Figure S13, in either the 2D COSY NMR of compound polyrotaxane 4 (Figure S13a) or compound polyrotaxane 5 (Figure S13b), we cannot observe the correlation signals other than their own coupling peaks. So, it could be confirmed that correlation signal in NOESY spectrum represents the coupling between the crown ether and the linear axis.

**Figure 3:**
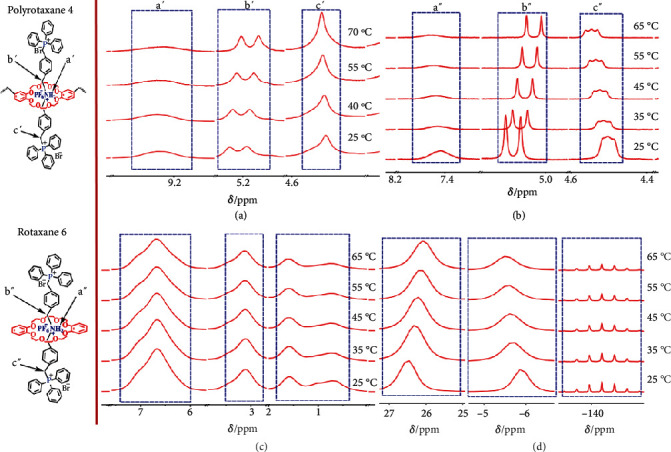
(a) Temperature-dependent liquid state ^1^H NMR of hydrogen-bonded polyrotaxane 4 (400 MHz, DMSO-*d6*). (b) Temperature-dependent liquid state ^1^H NMR of single molecular rotaxane 6 (400 MHz, DMSO-*d6*, 298 K). (c) Temperature-dependent solid-state ^1^H NMR spectra of rotaxane 6 (400 MHz). (d) Temperature-dependent solid-state ^31^P NMR spectra of rotaxane 6 (400 MHz).

The thermal-responsiveness of shuttling behavior could be reflected by the evolution of relative position between the crown ether host and the liner guest. At low temperatures, the linear guest has lower mobility and smaller shuttling range due to the hydrogen bonding. As a result, the sec-amine was mainly located in the caves of crown ether. With increasing temperature, the linear guest can easily overcome the energy barrier, enabling accelerated shuttling behavior. Other moieties in the linear guest (phenyl rings, benzyl methyl groups, etc.) can thus have a higher possibility to move into the caves of crown ether. As a verification of this assumption, temperature-dependent liquid-state ^1^H NMR analysis of hydrogen-bonded polyrotaxane 4 was conducted and depicted in Figure 3(a) and Figure S6. When temperature increases from 25 to 70°C, the NMR signal (a′) of -NH_2_^+^- shifted downfield from 9.2 to 9.3 ppm as a result of less shielding effect from the crown ether. Meanwhile, the NMR signals (b′) of methylene group shifted upfield from 5.21 to 5.15 ppm due to the higher possibility to coexist with crown ether in the same plane, suggesting that the linear guest can easily slip back and forth through the caves of the macrocyclic host. Besides, temperature-dependent liquid-state ^31^P NMR was also conducted. With increasing temperature from 25°C to 55°C, the phosphorus signal shifted upfield from 23.17 to 23.09 ppm, which is consistent with ^1^H NMR results (Figure S7).

In the above results, the shifting of proton signals from the linear guest was observed. However, the crown ether signals overlapped with -(CH_2_-CH_2_)_4_- signals in the backbone; thus, their shifting trend with increasing temperature could not be clearly observed. Therefore, a rotaxane 6 was synthesized as a model compound (Scheme S8) [24] and characterized by temperature-dependent ^1^H NMR (Figure 3(b) and Figure S10). The linear guest signals a^″^, b^″^, and c^″^ showed the same shifting trend as that of poly rotaxane results. These results confirmed that the polyrotaxane possesses temperature-responsive shuttling behavior in the liquid state, i.e., low energy barrier at high temperature leads to fast shuttling and extended motion range.

The next target is to prove the thermal-responsiveness of shuttling behavior in all solid-state since low humidity environment may occur in some applications like fuel cell [32]. Due to the low resolution of solid-state ^1^H NMR, the abovementioned rotaxane 6 was employed as the model compound. Its temperature-dependent solid-state ^1^H NMR was depicted in Figure 3(c) and Figure S11. With increasing temperature, the signals of aromatic protons (5.8-7.4 ppm) and aliphatic protons (2.6-3.6 ppm and 0.3-1.8 ppm) both showed changes in chemical shift and signal shape. However, due to the interference between multiple proton signals, peaks tend to widen and overlap with each other. Temperature-dependent ^31^P NMR of the above rotaxane 6 was further conducted to eliminate this effect (Figure 3(d) and Figure S12). The signal at 26.5 ppm was assigned to the phosphorus in the cationic group. With increasing temperature from 25°C to 65°C, apparent upfield shifting from 26.5 to 26.0 ppm was observed, suggesting an increasing shielding effect from the oxygen in the crown ether. These results confirmed that even in the solid-state, the rotaxane possesses temperature-responsive shuttling behavior.

**Figure 4:**
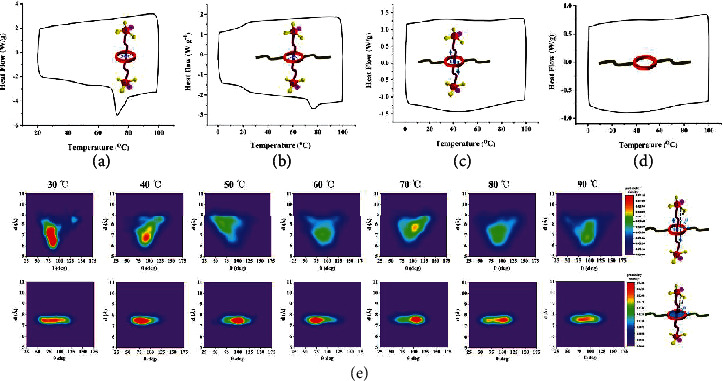
DSC profile of single molecular rotaxane (a), hydrogen-bonded polyrotaxane (b), free shuttling polyrotaxane (c), and poly crown ether host (d). (e) The joint probability density distribution of *d* and *θ* for polyrotaxane AEM and conventional tethered AEM. The variable *d* (*y*-axis) is defined as the distance from the cationic group to the crown ether plane; *θ* describes the angle between linear guest and crown ether plane. The range of probability density values for each color is shown in the legend; the red area represents high-density probability, and the green area represents low-density probability.

The above results suggest that there is a transition temperature for the polyrotaxane entity, above which the motion of linear guest will be significantly accelerated. To determine this transition temperature, differential scanning calorimetry (DSC) curve of the rotaxane 6 was recorded (Figure 4(a)). The curve showed a single endothermic peak over the range of 65-80°C, suggesting the activation temperature for overcoming the energy barrier and enabling the free shuttling behavior of linear guest. As for the hydrogen-bonded polyrotaxane 4, a higher transition temperature (70-80°C) was observed (Figure 4(b)) due to the extra steric hindrance effect from the high molecular weight and entanglement of polymer backbones. In Figure 4(c), the free shuttling polyrotaxane showed no endothermic peak due to the elimination of hydrogen bonding between crown ether host and linear guest, suggesting that the cationic group's high mobility could be achieved at a much lower temperature. Besides, no endothermic peak was detected for the poly(crown ether) host in the temperature range of 0 to 100°C (Figure 4(d)); thus, the possible interference from glass transition or melting of polymer backbones could be excluded.

Molecular dynamics simulation (Forcite module) of the polyrotaxane system was further conducted to intuitively elucidate the mobility and motion range of free shuttling cationic groups. The molecular structures were built and optimized under PCFF force field, and the angle-distance probability density distribution was plotted. As shown in Figure 4(e) and Figure S14, the distance from the cationic group to the crown ether plane is defined as vector *d* (*y*-axis), and the angle between linear guest and crown ether plane is defined as vector *θ* (*x*-axis). In order to simulate conventional tethered AEM, a constant restraint of 10 kcal/mol/Å^2^ was applied between crown ether and the -NH_2_^+^- group to simulate the covalent bonding (Figure S14b). The color from blue to red reflects the increased probability density distribution of a particular relative positive between cationic groups and the crown ether plane. At low temperatures, both the conventional tethered AEM structure and the as-designed polyrotaxane structure showed *θ* in the range of 50-130^o^, suggesting similar oscillation behavior of the cationic groups. However, the polyrotaxane structure showed a *d* value of 5.5-9 Å, which is in sharp contrast to the conventional tethered structure (*d* = 7-8 Å). This suggests the linear guest can shuttle back and forth through caves of crown ether. The oscillation and shuttling behavior of phosphonium cations originating from its nontethered nature would ensure improved mobility and extended motion range to promote bulky anions' conduction.

With increasing temperatures, the motion behavior of conventional AEM shows no noticeable change, with a narrow range of relative positions as well as a highly concentrated possibility density in the center. These results suggest that the ion-conducting groups are mainly fixed at one particular position due to the confinement of the covalent bond. In sharp contrast, the polyrotaxane AEM shows both increased scope of relative position and more even distribution of possibility density due to the unique shuttling characteristic of poly-rotaxane. In addition, evident temperature responsiveness was observed. Above 50°C, the motion range of phosphonium cations increased obviously. This free shuttling behavior with an extensive motion range is believed to refine bulk anions' conduction mechanism, by providing extra solvation shell fluctuation [21] and reducing the energy barrier when transferring between two cationic groups [19].

**Figure 5:**
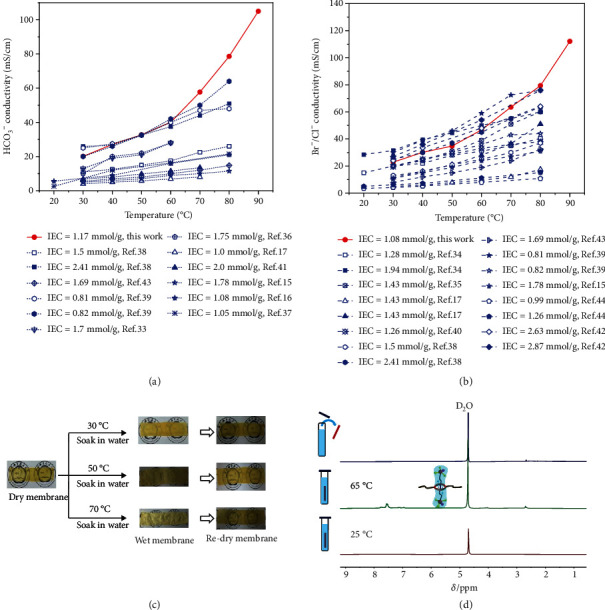
(a) Comparison of temperature-dependent HCO_3_^−^ conductivity of polyrotaxane AEM and conventional tethered AEMs. (b) Comparison of temperature-dependent Br^−^ conductivity of polyrotaxane AEM and conventional tethered AEMs. (c) Change of physical appearance of polyrotaxane AEM when soaked in water at different temperatures. (d) ^1^H NMR spectra of polyrotaxane AEM soaked in D_2_O at different temperatures.

AEMs were prepared from the free shuttling polyrotaxane 5 via solution casting method. Its properties including ion exchange capacity, water uptake, swelling ratio, and Br^−^/HCO_3_^−^/OH^−^ conductivity are summarized in Table S1, Table S2, and Figure S15. The mechanical strength and thermal stability were also measured (Figure S16). Results showed that mechanical properties (tensile strength and elongation at break) of the hydrated membrane are improved with the increasing temperature (Figure S16a), and the AEM possesses good thermal stability up to 150°C with sufficient for practical application (Figure S16b). Firstly, the favorable advantages from the free shuttling phosphonium cations were demonstrated by comparing the conductivity of the polyrotaxane-based AEM and conventional tethered AEMs (Figures 5(a) and 5(b)) [15–17, 33–44]. For conventional AEMs, the HCO_3_^−^ and Br^−^conductivities showed an increasing linear trend with temperature. Meanwhile, the conductivity-temperature plot of polyrotaxane AEMs shows a distinct two-segment characteristic. From 30°C to 60°C, the conductivity increased slowly with temperature, from 20.1 to 40.1 mS cm^−1^ for HCO_3_^−^ and 22.9 to 46.7 mS cm^−1^ for Br^−^. Above the transition temperature (65°C), the accelerated shuttling of phosphonium cations yielded a dramatic leap in conductivity, i.e., 105 mS cm^−1^ for HCO_3_^−^ and 112.1 mS cm^−1^ for Br^−^ at 90°C, by enabling higher anion mobility and better anion dissociation. Note that this good conductivity was achieved at a low ion exchange capacity of 1.17 mmol/g for HCO_3_^−^ and 1.08 mmol/g for Br^−^ (IEC is defined as mmol of ion-conducting groups in per gram of dried membranes, Table S1).

Apart from the dramatically improved conductivity, the hydrated AEM also shows a thermal triggered appearance transition. As shown in Figure 5(c), the dry transparent membrane in the dehydration state became opaque after soaked in water at 70°C for 2 hours, suggesting the dissolution of the linear guest in water. Thus, the remaining hydrophobic polymer backbone became incompatible with the aqueous linear guest, and macroscopic phase separation occurred. Interestingly, this transition behavior is reversible when the membrane became transparent again after it was dried. This interesting thermal triggered appearance transition further proved the temperature responsiveness and reversibility of shuttling behavior. To exclude the water effect, the dry membrane was heated at 70°C for 2 hours, and the result is shown in Figure S18a. The results show that there is no obvious appearance change of membrane. We also tested the SEM of the dry membrane, as shown in Figure S18b and Figure S18c. It is noteworthy that the dry membrane displayed quite a compact structure with no defect, which is coherent to its transparent and dense appearance as shown in Figure S18a.

The above assumption was confirmed by ^1^H NMR analysis. Figure 5(d) shows the ^1^H NMR spectra of the membrane/D_2_O mixture. When the temperature increased from 25°C to 60°C, the grafted chain's proton signals appeared. Moreover, all the proton signals disappeared when the membrane was removed from the D_2_O. It indicates that the dissolution of the mobile guest chains in the water channel is temperature-dependent and that diffusion loss from the poly(crown ether) host does not occur. Therefore, the polyrotaxane structure resembled a polymer blend with hydrophilic cationic groups that can probably dissolve in the water channel and possess high mobility to accelerate bulky anion's conduction.

## 3. Discussion

In conclusion, a polyrotaxane system featured with free shuttling phosphonium cations as ion-conducting group was assembled to address bulky anions' low conductivity. The temperature responsiveness and reversibility of shuttling behavior were proved by temperature-dependent solid-state NMR and molecular dynamics simulation. This exceptional free-shuttling anion carrier allows promoted anion dissociation, hydration, and mobility, thus yielding an excellent anion conductivity of 105 mS cm^−1^ for HCO_3_^−^ and 112.1 mS cm^−1^ for Br^−^ at a low ion exchange capacity. Broad applications of this unique polyrotaxane system in ion separation processes are currently in progress.

## 4. Materials and Methods

### 4.1. Materials

Hydrobromic acid, triphenylphosphine, and magnesium sulfate were purchased from Sinopharm Chemical Reagent Co., Ltd. Eaton's reagent, diphenyl ether (99%), sebacic acid (98%), methyl-4-formylbenzoate (99%), ammonium hexafluorophosphate (98%), and lithium aluminum hydride (LiAlH_4_) (97%) methyl-4-(aminomethyl) benzoate hydrochloride were purchased from Shanghai Energy-Chemical Co. Ltd. Dibenzo-24-crown-8 was purchased from Tokyo Chemical Industry (TCI). All reagents were used as received.

### 4.2. Preparation of Poly(Crown Ether) 1

Dibenzo-24-crown-8 (1 mmol), sebacic acid (4 mmol), and diphenyl ether (3 mmol) were dissolved in Eaton's reagent (12.8 mL), and the mixture was heated to 40°C and stirred for 24 h. After the reaction was stirred for 24 h, it was poured into water. The slightly yellow fibers that formed were filtered off and washed with water. After the fibers were dried, compound 1d was obtained as a white fiber-like polymer, 2a (98%).

### 4.3. Preparation of Compound 2c

Compound 2a (1 mmol) and compound 2a (1 mmol) were dissolved in dry toluene (60 mL), and the mixture was heated under reflux in an argon atmosphere for 24 h using a Dean-Stark apparatus. After the reaction mixture cooled, the solvents were removed under vacuum, and the remaining components were washed with ethanol (3 × 30 mL). Compound 2c was isolated as a white solid (85%).

### 4.4. Preparation of Compound 2d

A solution of compound 2c (1 equiv) in dry THF was cooled to 0°C, and powdered LiAlH_4_ (6 equiv) was added to the solution over a period of 1 h. The mixture was then warmed to room temperature and heated under reflux in an argon atmosphere overnight. After the reaction was stirred overnight, it was cooled to 0°C. Sodium sulfate decahydrate (2 equiv) was then carefully added to the flask. The mixture was stirred for 1 h and filtered and washed with THF. The filtrate was collected and dried (with MgSO_4_), and the solvent was removed under vacuum to give compound 2d as a yellow liquid (70%).

### 4.5. Preparation of Compound 2

Compound 2d (1 mmol) was dissolved in 30 mL of HBr (48%), and the resulting mixture was heated to 100°C and stirred for 24 h. Then, it was cooled to room temperature and filtered and washed with water. The white solid was dissolved in a saturated, aqueous NH_4_PF_6_ solution and stirred for 24 h. The reaction mixture was again filtered and washed with water to give compound 2 as a white solid (85%).

### 4.6. Preparation of Polyrotaxane 4

Compound 1 (1 mmol) and compound 2 (1 mmol) were dissolved in CH_3_CN/CHCl_3_ (30 mL, 1 : 2); the mixture was stirred at room temperature for 24 h. Then, triphenylphosphine (2.5 mmol) was added to the reaction solution and left to stir at room temperature for 48 h. After the mixture was stirred for 48 h, it was poured into diethyl ether and stirred. The yellow fibers that formed were filtered off, wash with diethyl ether, and dried under flowing air to give compound 4.

### 4.7. Preparation of Membrane 4·Br^−^, 5·OH^−^, 5·HCO_3_^−^

Polyrotaxane 4 was dissolved in CH_3_CN to form a 5 wt% casting solution that was cast onto a tetrafluoroethylene plate. The cast films were heated at 30°C to remove the solvent. After the drying process, flexible, transparent, and yellow-tinged polyrotaxane AEMs 4·Br^−^ (with thickness of 50 ± 5 *μ*m) were obtained. All membranes were fully converted to the HCO_3_^−^ and OH^−^ form via immersion in aqueous KHCO_3_ (1 mol·L^−1^) and KOH (1 mol·L^−1^) solution at room temperature for 24 h, followed by thorough washing and storage in sealed sample bottles (full of deionized water). This membrane was denoted polyrotaxane AEMs 5·HCO_3_^−^ and 5·OH^−^.

### 4.8. Preparation of Rotaxane 6

Dibenzo-24-crown-8 (1 mmol) and compound 2 (1.5 mmol) were dissolved in CH_3_CN/CHCl_3_ (20 mL, 1 : 1); the mixture was stirred at room temperature for 1 h. Then, triphenylphosphine (4 mmol) was added to the solution; the reaction was then left to stir at room temperature for 24 h. The resulting white precipitate was filtered off and washed with CH_2_Cl_2_. The organic layer was removed, and the resulting solid redissolved in CH_2_Cl_2_ and any insoluble material removed by filtration. Et_2_O was then added to the CH_2_Cl_2_ solution; the resulting white precipitate was filtered and wash with more Et_2_O to give rotaxane 6.

### 4.9. Molecular Dynamics (MD) Simulations

Molecular dynamics simulations were performed using Forcite module. Molecular structures were built and optimized under PCFF force field. We performed 400 ps dynamics of AEM under NVT ensemble with different temperature of 303-363 K. The Nosé–Hoover–Langevin thermostat (Qratio of 0.01) was used to control temperature, with 1 fs time step. To elucidate the difference in mobility and motion range of the free shuttling AEM and conventional tethered AEM, the angle-distance probability density distribution was plotted. As shown in Figure S14a, the center mass of rotaxane was defined as point A, the P atom in the phosphonium cations was defined as point P, and O atom in crown ether was defined as point O. The angle (*θ*) was defined as P-A-O, and the distance (*d*) was defined as P-A. In order to simulate conventional tethered AEM, a constant restraint of 10 kcal/mol/Å^2^ was applied between crown ether and the -NH_2_^+^- group to simulate the covalent bonding (Figure S14b). In the probability density distribution profile (Figure S14c), red zone indicates high density probability, and green zone indicates low density probability.

## Data Availability

All data needed to evaluate the conclusions in the paper are present in the paper and/or the Supplementary Information.

## Supplementary Materials

Supplementary Materials Supplementary. Scheme S1: synthesis of poly (crown ether) 1. Figure S1: ^1^H NMR spectrum of poly (crown ether) 1 (400 MHz, CDCl_3_). Scheme S2: synthesis of compound 2c. Figure S2: ^1^H NMR spectrum of compound 2c (400 MHz, CDCl_3_). Scheme S3: synthesis of compound 2d. Figure S3: ^1^H NMR spectrum of compound 2d (400 MHz, DMSO-d6). Scheme S4: synthesis of compound 2. Figure S4: ^1^H NMR spectrum of compound 2 (400 MHz, DMSO-d6). Scheme S5: synthesis of polyrotaxane 4. Figure S5: ^1^H NMR spectrum of polyrotaxane 4 (400 MHz, DMSO-d6). Figure S6: temperature-dependent ^1^H NMR spectrum of polyrotaxane 4 (400 MHz, DMSO-d6). Figure S7: temperature-dependent ^31^P NMR spectrum of compound 4 (400 MHz, DMSO-d6). Scheme S6: preparation of polyrotaxane AEMs 5·HCO_3_-. Scheme S7: preparation of polyrotaxane AEMs 5·OH-. Figure S8: ^1^H NMR spectrum of polyrotaxane AEM 5·OH- (400 MHz, DMSO-d6). Scheme S8: synthesis of rotaxane 6. Figure S9: ^1^H NMR spectrum of rotaxane 6 (400 MHz, DMSO-d6). Figure S10: temperature-dependent ^1^H NMR spectrum of rotaxane 6 (400 MHz, DMSO-d6). Figure S11: temperature-dependent solid-state ^1^H NMR spectra of rotaxane 6 (400 MHz). Figure S12: temperature-dependent solid-state ^31^P NMR spectra of rotaxane 6 (400 MHz). Figure S13: (a) COSY 2D NMR of hydrogen-bonded polyrotaxane 4 (400 MHz, DMSO-d6); (b) COSY 2D NMR of free shuttling polyrotaxane 5 (400 MHz, DMSO-d6). Figure S14: (a) Molecular structure of polyrotaxane AEM in molecular dynamics system; (b) molecular structure of conventional tethered AEM in molecular dynamics system; (c) the joint probability density distribution of *d* and *θ* for polyrotaxane AEM and conventional tethered AEM. Table S1: IEC values of polyrotaxane AEMs. Table S2: WU and DSR of polyrotaxane AEMs. Figure S15: temperature-dependent Br-/HCO_3_-/OH- conductivity of polyrotaxane AEMs. Figure S16: (a) Mechanical properties of the AEMs 4·Br-; TGA curves of the AEMs 4·Br-, 5·OH-, and 5·HCO_3_-. Figure S17: schematic illustration of hydroxide ion (OH-) transport. Figure S18: (a) Change of physical appearance of polyrotaxane AEM was heated at 70°C for 2 h; (b) surface SEM images of polyrotaxane AEM; (c) cross-section SEM images of polyrotaxane AEM.
