# Supplementary material for: Fast Bulky Anion Conduction Enabled by Free Shuttling Phosphonium Cations
Source: Research (Wash D C). 2021 Aug 31;2021:9762709. doi: 10.34133/2021/9762709 (PMC8426568; doi:10.34133/2021/9762709)
Supplement: Supplementary Materials — Supplementary. Scheme S1: synthesis of poly (crown ether) 1. Figure S1: 1H NMR spectrum of poly (crown ether) 1 (400 MHz, CDCl3). Scheme S2: synthesis of compound 2c. Figure S2: 1H NMR spectrum of compound 2c (400 MHz, CDCl3). Scheme S3: synthesis of compound 2d. Figure S3: 1H NMR spectrum of compound 2d (400 MHz, DMSO-d6). Scheme S4: synthesis of compound 2. Figure S4: 1H NMR spectrum of compound 2 (400 MHz, DMSO-d6). Scheme S5: synthesis of polyrotaxane 4. Figure S5: 1H NMR spectrum of polyrotaxane 4 (400 MHz, DMSO-d6). Figure S6: temperature-dependent 1H NMR spectrum of polyrotaxane 4 (400 MHz, DMSO-d6). Figure S7: temperature-dependent 31P NMR spectrum of compound 4 (400 MHz, DMSO-d6). Scheme S6: preparation of polyrotaxane AEMs 5·HCO3-. Scheme S7: preparation of polyrotaxane AEMs 5·OH-. Figure S8: 1H NMR spectrum of polyrotaxane AEM 5·OH- (400 MHz, DMSO-d6). Scheme S8: synthesis of rotaxane 6. Figure S9: 1H NMR spectrum of rotaxane 6 (400 MHz, DMSO-d6). Figure S10: temperature-dependent 1H NMR spectrum of rotaxane 6 (400 MHz, DMSO-d6). Figure S11: temperature-dependent solid-state 1H NMR spectra of rotaxane 6 (400 MHz). Figure S12: temperature-dependent solid-state 31P NMR spectra of rotaxane 6 (400 MHz). Figure S13: (a) COSY 2D NMR of hydrogen-bonded polyrotaxane 4 (400 MHz, DMSO-d6); (b) COSY 2D NMR of free shuttling polyrotaxane 5 (400 MHz, DMSO-d6). Figure S14: (a) Molecular structure of polyrotaxane AEM in molecular dynamics system; (b) molecular structure of conventional tethered AEM in molecular dynamics system; (c) the joint probability density distribution of d and θ for polyrotaxane AEM and conventional tethered AEM. Table S1: IEC values of polyrotaxane AEMs. Table S2: WU and DSR of polyrotaxane AEMs. Figure S15: temperature-dependent Br-/HCO3-/OH- conductivity of polyrotaxane AEMs. Figure S16: (a) Mechanical properties of the AEMs 4·Br-; TGA curves of the AEMs 4·Br-, 5·OH-, and 5·HCO3-. Figure S17: schematic illustration of hydroxide ion (OH-) transp [file 9762709.f1.docx]

Fast Bulky Anion Conduction Enabled by Free Shuttling Phosphonium Cations

Xiaolin Ge,^1,†^ Yubin He,^1,†^ Kaiyu Zhang,^1^ Xian Liang, ^1,2^ Chengpeng Wei,^1^ Muhammad A. Shehzad,^1^ Wanjie Song,^1^ Zijuan Ge,^1^ Geng Li,^1^ Weisheng Yu,^1^ Liang Wu,^1,^* Tongwen Xu.^1,^*

^1^ CAS Key Laboratory of Soft Matter Chemistry, Collaborative Innovation Center of Chemistry for Energy Materials, School of Chemistry and Materials Science, University of Science and Technology of China, 96 Jinzhai Road, Hefei, Anhui 230026, China.

^2^ School of Chemistry and Material Engineering, Huainan Normal University, Huainan, Anhui 232001, China.

* Correspondence author. E-mail: twxu@ustc.edu.cn, liangwu8@ustc.edu.cn

† Equally contributed to this work

**1. Methods**

**1.1 Characterizations**

^1^H NMR ,^31^P NMR , NOESY spectra, COSY spectra and SSNMR were recorded on a Bruker Avance III 400 MHz spectrometer. Chemical shifts are reported in ppm relative to the signals corresponding to the residual non-deuterated protons in NMR solvents (CDCl_3_: δ 7.26 ppm, DMSO-d_6_: δ 2.53 ppm, D_2_O: δ 1.56 ppm). Tensile strength and elongation at break of the membranes were measured using a Q800 dynamic mechanical analyser (DMA, TA Instruments) at a stretch rate of 0.5 N min-1 under air atmosphere. Thermal stability of the membrane was characterized by a Q5000 thermo gravimetric analyzer (TGA, TA Instruments) under air flow with a temperature increase rate of 10 °C per minute. Transmission electron microscopy were detected through JEM 2100F field emission transmission electron microscope (TEM, JEOL Ltd., Japan). Stain the AEM samples with iodide anions, then using LEICA UC6+UC7 ultramicrotome to selected the dried samples into ~60 nm thick slices and coated on a Cu grid.

**1.2 Differential Scanning Calorimetry Thermograms (DSC).**

DSC measurements were performed on a DSC Q2000 thermal analysis system (TA instruments, USA). Polymer **1**, Polyrotaxane **4**, AEM **5** and Rotaxane **6** samples were initially heated from 20 to 100 °C at a heating rate of 10 °C min^-1^ under a nitrogen atmosphere, held at 100 °C for 3 min, and cooled to 20 °C at a rate of 10 °C min^-1^.

**1.3 Ion exchange capacity (IEC).**

IEC values were measured by titration method. The membrane samples were firstly immersed in NaCl (1 M) aqueous solution for 24 h. Afterwards, the samples were thoroughly washed with DI water and then immersed in DI water for 12 h. DI water was changed every half hour. Subsequently, the membrane samples were immersed in aqueous Na_2_SO_4_ solution (0.5 M) for 24 h. The Cl^-^ ions released from the membrane were titrated with aqueous AgNO_3_ solution (C*_AgNO3_* = 0.01 M) using K_2_CrO_4_ as indicator. The IEC values were calculated from the amount of AgNO_3_ consumed (V*_AgNO3_*) and the weight of dry membranes samples （m *_dry_*）.

$\mathrm{IEC}\left( \frac{\mathrm{mmol}}{g} \right)=\frac{V_{{AgNO}_{3}}\times C_{{AgNO}_{3}}}{m_{dry}}$……………...……………. (S1)

**1.4 Water uptake (WU) and dimensional swelling ration (DSR).**

WU and DSR was determined by measuring the weight and length changes of AEM **5** before and after hydration. Membrane samples (4 cm × 1 cm) were weighed after being dried at 80 °C under vacuum for 12 h and, then, immersed in deionized water at 60 °C for 24 h. The hydrated samples were then wiped with tissue paper to remove excess surface water, and their mass were quickly measured. WU and DSR was calculated as follows:

$\mathrm{WU}\left( \% \right)=\frac{100\%\times(W_{wet}-W_{dry})}{W_{dry}}$ (S2)

$DSR(\%)=\frac{100\%\times(L_{wet}-L_{dry})}{L_{dry}}$ (S3)

where W_wet_ and W_dry_ were the mass of the hydrated and dry AEM sample, respectively, L_wet_ and L_dry_ were the lengths of hydrated and dehydrated AEM sample, respectively.

**1.5 Ion conductivity.** The Br^-^, HCO_3_^-^, OH^-^ conductivity was measured following a standard four-point probe technique using an Autolab PGSTAT 30 (Eco Chemie, Netherland) potentiostat alongside a Teflon conductivity cell. The membrane (4 cm×1 cm) in the Br^-^, HCO_3_^-^, OH^-^ form was mounted onto the cell, screwed and fully hydrated in water at the desired temperature before the test. The testing procedure was carried out in galvanostatic mode (monitored using a Bode plot) with a current amplitude of 0.1 mA over a frequency range of 1 MHz to 50 Hz. The corresponding resistance was obtained from a Nyquist plot. The conductivity (σ) along the membrane was calculated according to the following equation:

$\sigma=\frac{L}{RWd}$ (S4)

where R is the absolute ohmic resistance of the AEM sample, L is the distance between potential-sensing electrodes; W is width of the membrane, *d* is thickness of the membrane, respectively.

**2. Synthesis of polyrotaxane and preparation of membrane.**

The synthesis procedures used to prepare **1**, **2c**, **2d**, **2**, **4**, **5•OH^-^, 5•HCO_3_^-^, 6** are shown in **Scheme S1, S2, S3, S4,** **S5, S6, S7** and **S8**. Analytical thin-layer chromatography (TLC) was performed on glass sheets pre-coated with the silica gel 60-F254. The ^1^H NMR spectra are recorded in ppm.

**2.1 General procedure for the synthesis of poly(crown ether) 1**

^1^H NMR (400 MHz, CDCl_3_) δ 7.97 (dd, J = 19.9, 8.2 Hz, 8H), 7.55 (ddd, J = 22.1, 15.5, 7.4 Hz, 4H), 7.39 (t, J = 7.7 Hz, 3H), 7.20 (t, J = 7.3 Hz, 2H), 7.07 (dd, J = 8.5, 3.9 Hz, 8H), 6.99 (t, J = 5.9 Hz, 3H), 6.93 – 6.83 (m, 2H), 4.68 – 4.16 (m, 14H), 4.06 – 3.56 (m, 10H), 3.24 – 3.13 (m, 3H), 3.07 (t, J = 5.1 Hz, 1H), 2.93 (dd, J = 16.5, 8.6 Hz, 10H), 2.34 (q, J = 7.3 Hz, 2H), 1.77 – 1.25 (m, 48H).


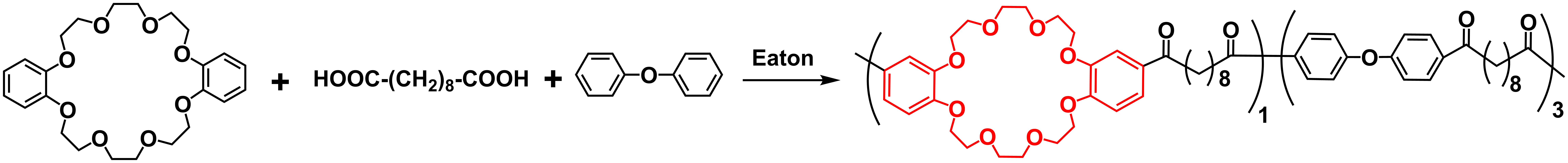


**Scheme S1**. Synthesis of **poly (crown ether) 1.**


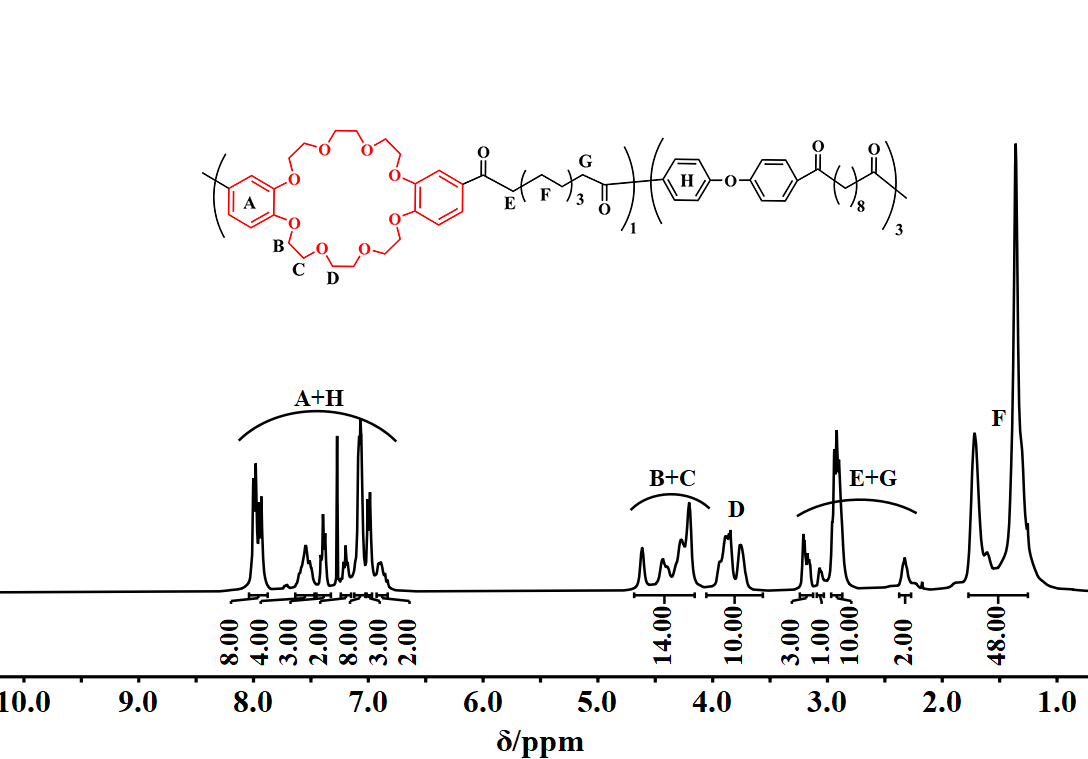


**Figure S1** ^1^H NMR spectrum of **poly (crown ether) 1** (400 MHz, CDCl_3_)

**2.2** **General procedure for the synthesis of compound 2c.**

^1^H NMR (400 MHz, CDCl_3_) δ 8.46 (s, 1H), 8.10 (d, J = 8.3 Hz, 2H), 8.03 (d, J = 8.2 Hz, 2H), 7.86 (d, J = 8.3 Hz, 2H), 7.43 (d, J = 8.0 Hz, 2H), 4.90 (s, 2H), 3.93 (d, J = 9.6 Hz, 6H).


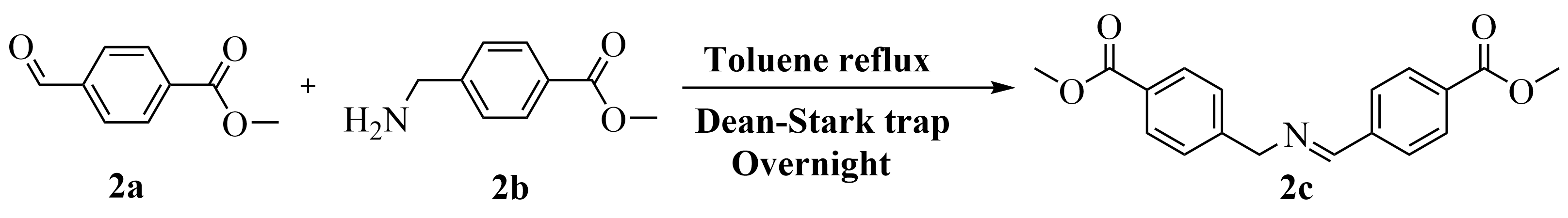


**Scheme S2**. Synthesis of compound **2c**.


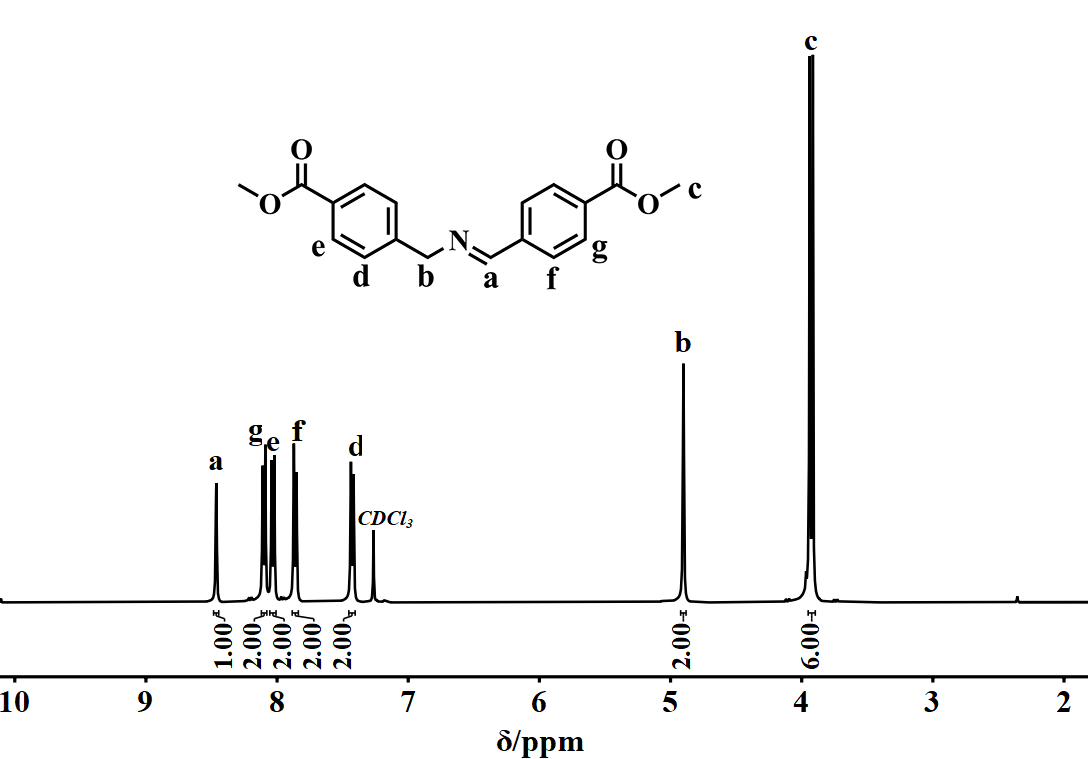


**Figure S2.** ^1^H NMR spectrum of compound **2c** (400 MHz, CDCl_3_)

**2.3** **General procedure for the synthesis of compound 2d.**

^1^H NMR (400 MHz, DMSO-*d_6_*) δ 7.37 – 7.26 (m, 8H), 5.24 (t, J = 5.7 Hz, 2H), 4.52 (d, J = 4.8 Hz, 4H), 3.68 (s, 4H), 3.58 – 3.35 (m, 1H).


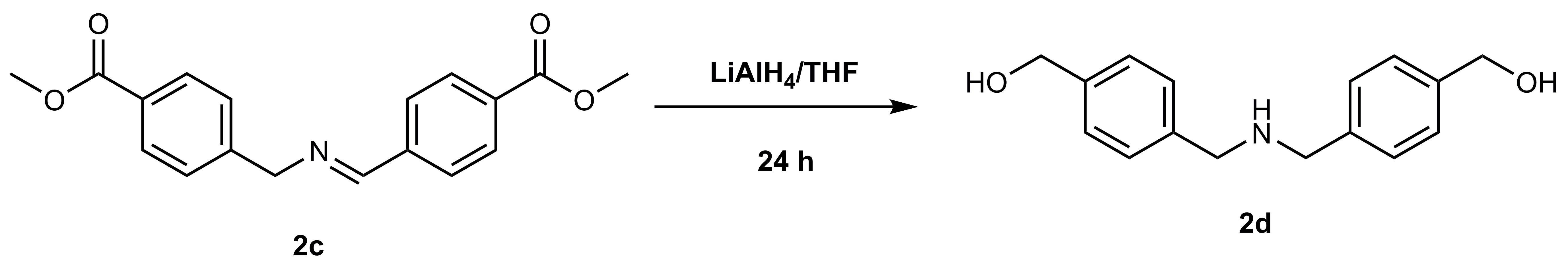


**Scheme S3**. Synthesis of compound **2d**


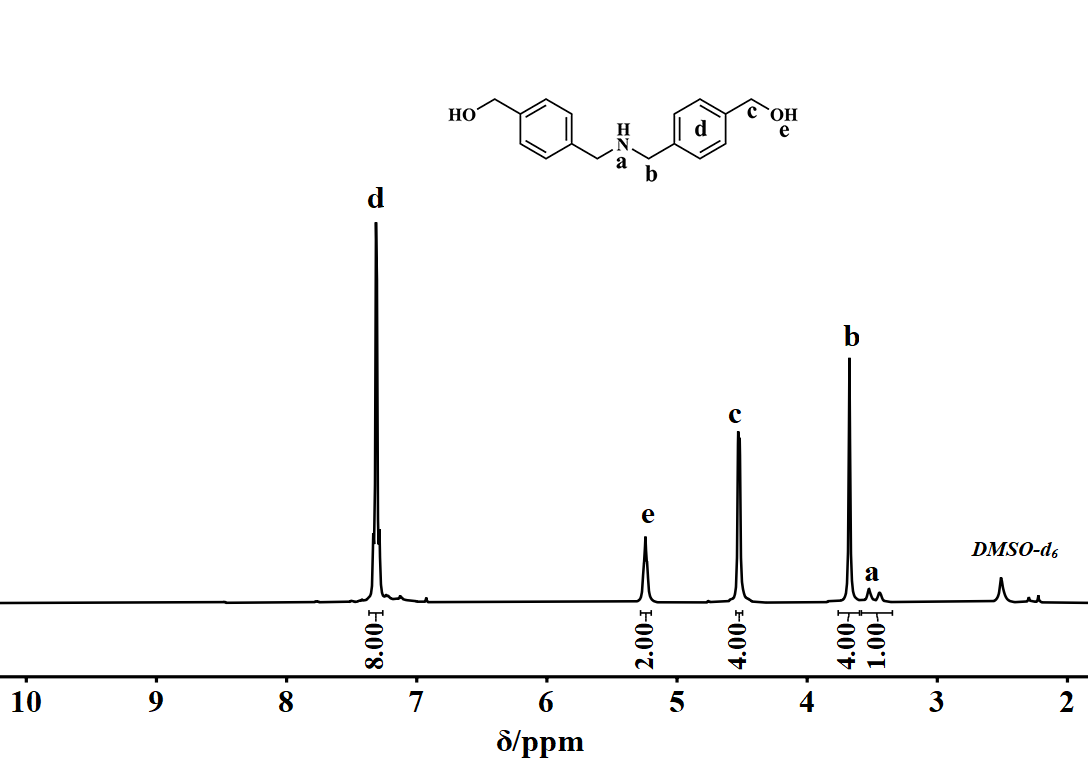


**Figure S3.** ^1^H NMR spectrum of compound **2d** (400 MHz, DMSO-*d_6_*)

**2.4** **General procedure for the synthesis of compound 2.**

^1^H NMR (400 MHz, DMSO-*d_6_*) δ 9.19 (s, 2H), 7.52 (p, J = 8.4, 7.8 Hz, 8H), 4.74 (s, 4H), 4.19 (d, J = 5.9 Hz, 4H).


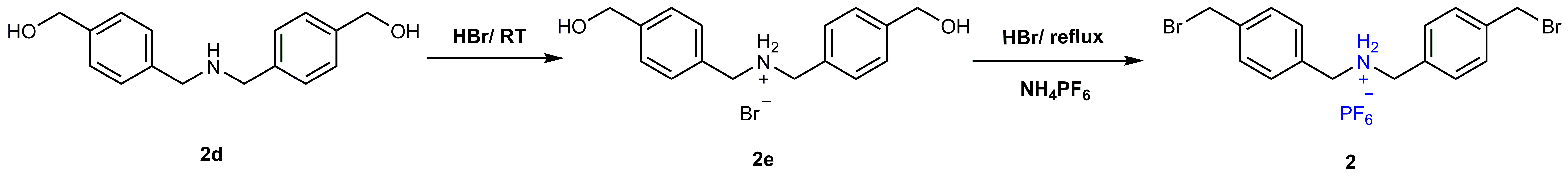


**Scheme S4.** Synthesis of compound **2**


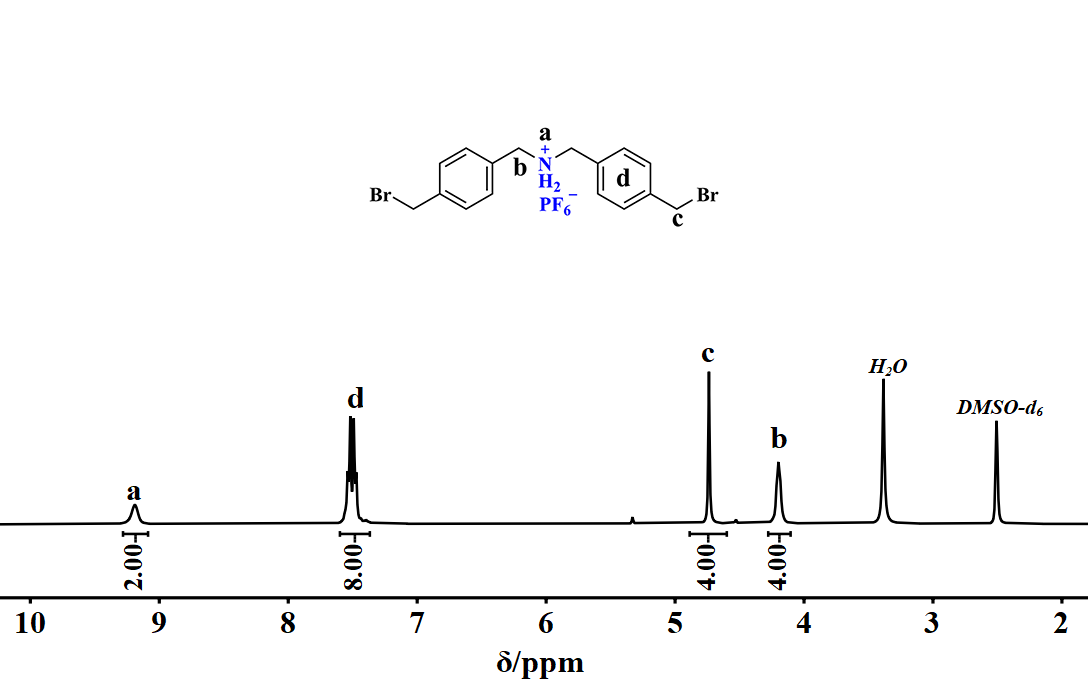


**Figure S4.** ^1^H NMR spectrum of compound **2** (400 MHz, DMSO-*d_6_*)

**2.5** **General procedure for the synthesis of Polyrotaxane 4.**

^1^H NMR (400 MHz, DMSO-*d_6_*) δ 9.25 (s, 2H), 8.28 (s, 4H), 8.11 – 7.85 (m, 10H), 7.82 – 7.59 (m, 15H), 7.63 – 7.30 (m, 15H), 7.36 – 6.50 (m, 24H), 5.22 (d, J = 15.9 Hz, 4H), 4.54 (d, J = 12.8 Hz, 4H), 4.18 – 4.03 (m, 10H), 3.77 – 3.57 (m, 14H), 2.34 (s, 5H), 2.16 (d, J = 8.3 Hz, 6H), 1.92 – 1.87 (m, 6H), 1.40 (d, J = 119.7 Hz, 48H).


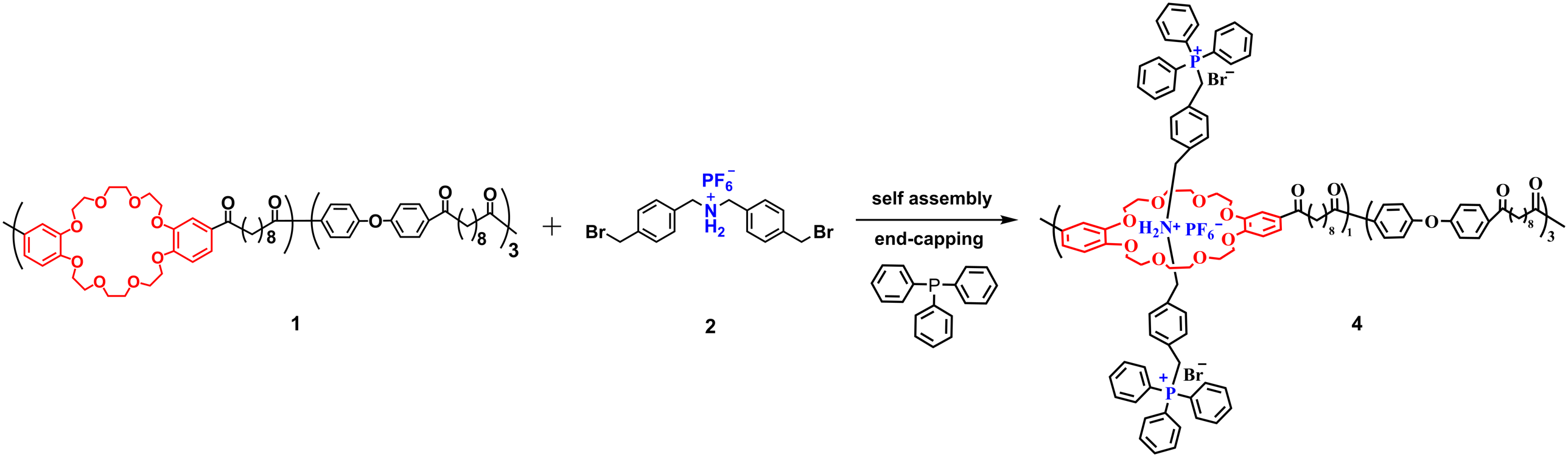


**Scheme S5.** Synthesis of polyrotaxane **4**


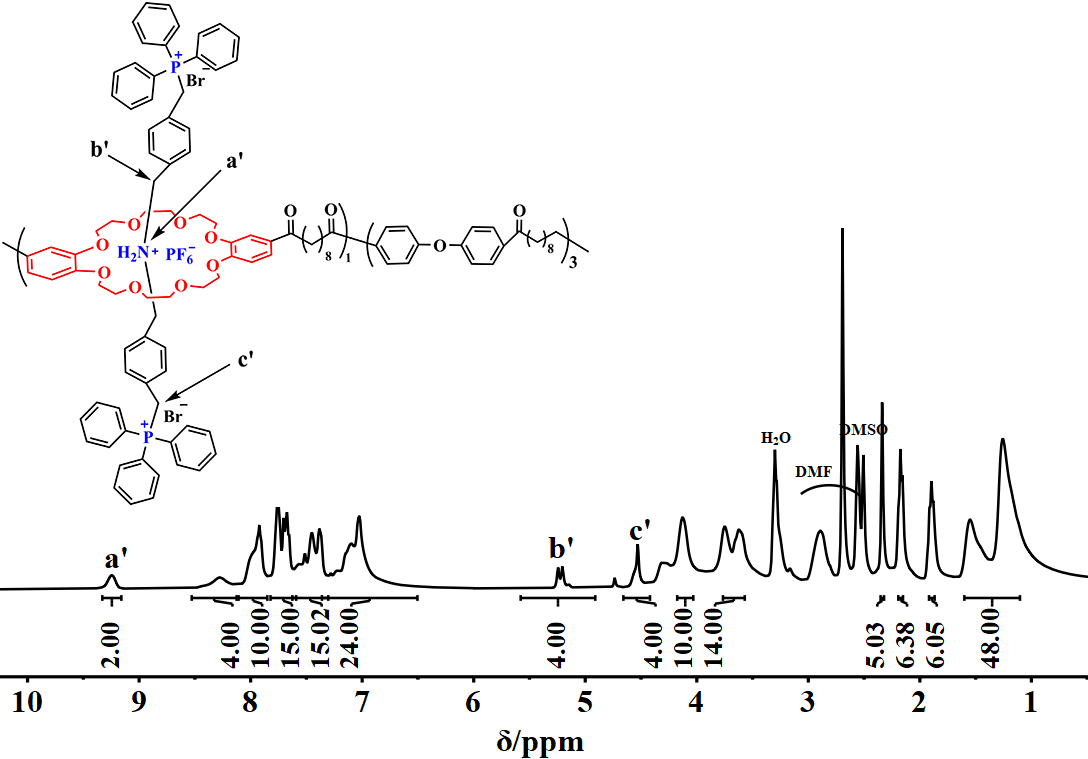


**Figure S5.** ^1^H NMR spectrum of polyrotaxane **4** (400 MHz, DMSO-*d_6_*)


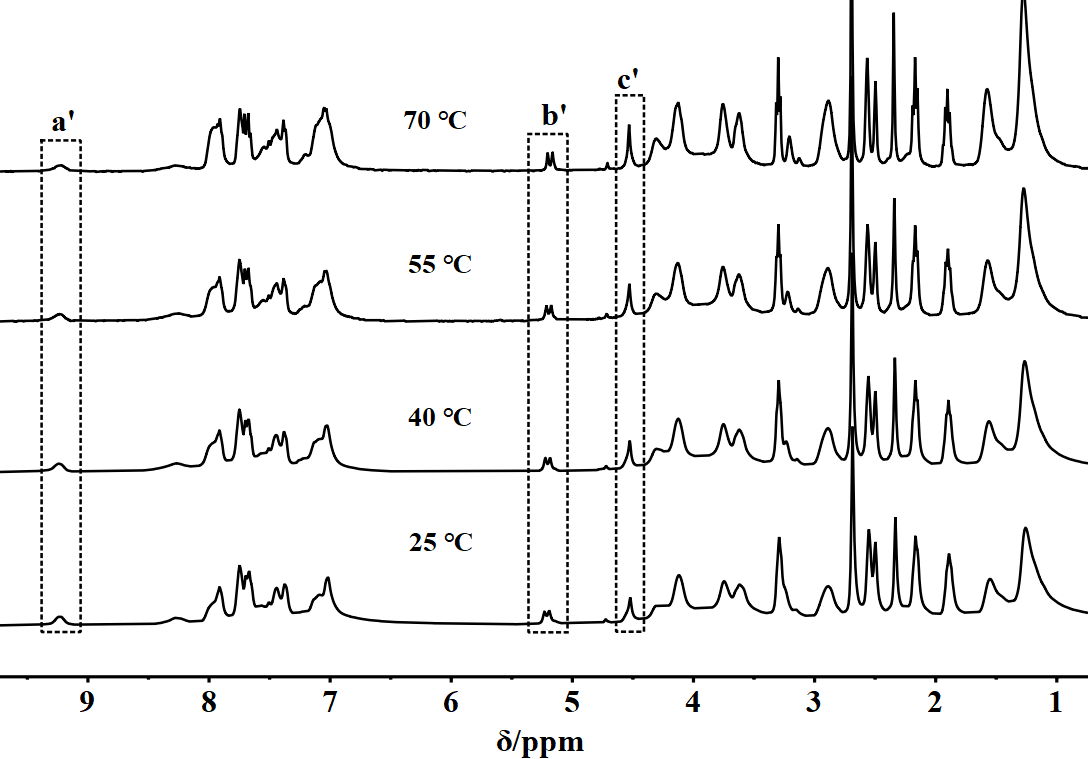


**Figure S6.** Temperature dependent ^1^H NMR spectrum of polyrotaxane **4** (400 MHz, DMSO-*d_6_*)


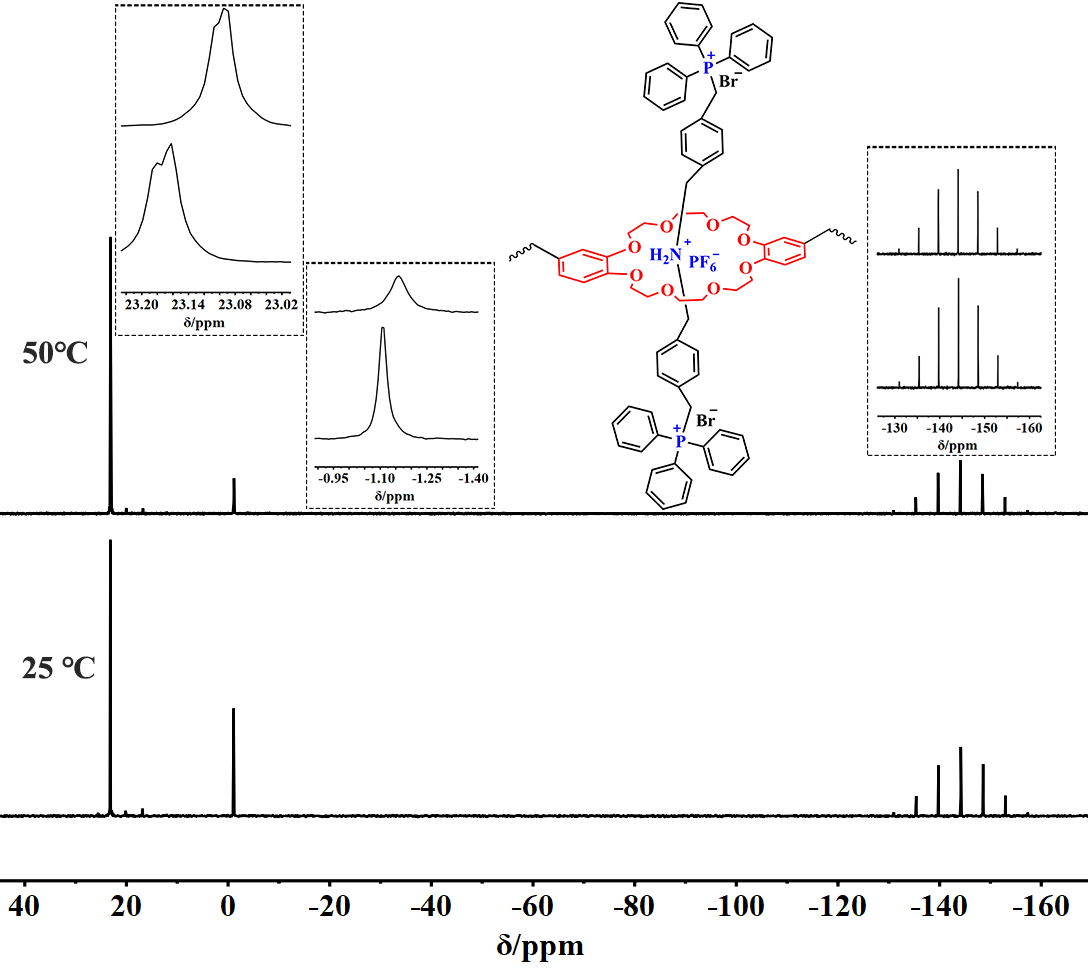


**Figure S7.** Temperature dependent ^31^P NMR spectrum of compound **4** (400 MHz, DMSO-*d_6_*)

**2.6** **General procedure for preparation of membrane 4•Br^-^, 5•OH^-^, 5•HCO_3_^-^**.

^1^H NMR (400 MHz, DMSO-*d_6_*) δ 7.94 (t, J = 21.4 Hz, 14H), 7.78 – 7.63 (m, 21H), 7.51 – 7.34 (m, 10H), 7.27 – 6.94 (m, 24H), 5.31 (d, J = 16.4 Hz, 1H), 5.20 (d, J = 15.9 Hz, 4H), 4.52 (s, 4H), 3.81 – 3.59 (m, 24H), 2.87 (s, 6H), 2.35 (s, 4H), 2.28 – 2.09 (m, 6H), 1.36 (d, J = 95.2 Hz, 48H).


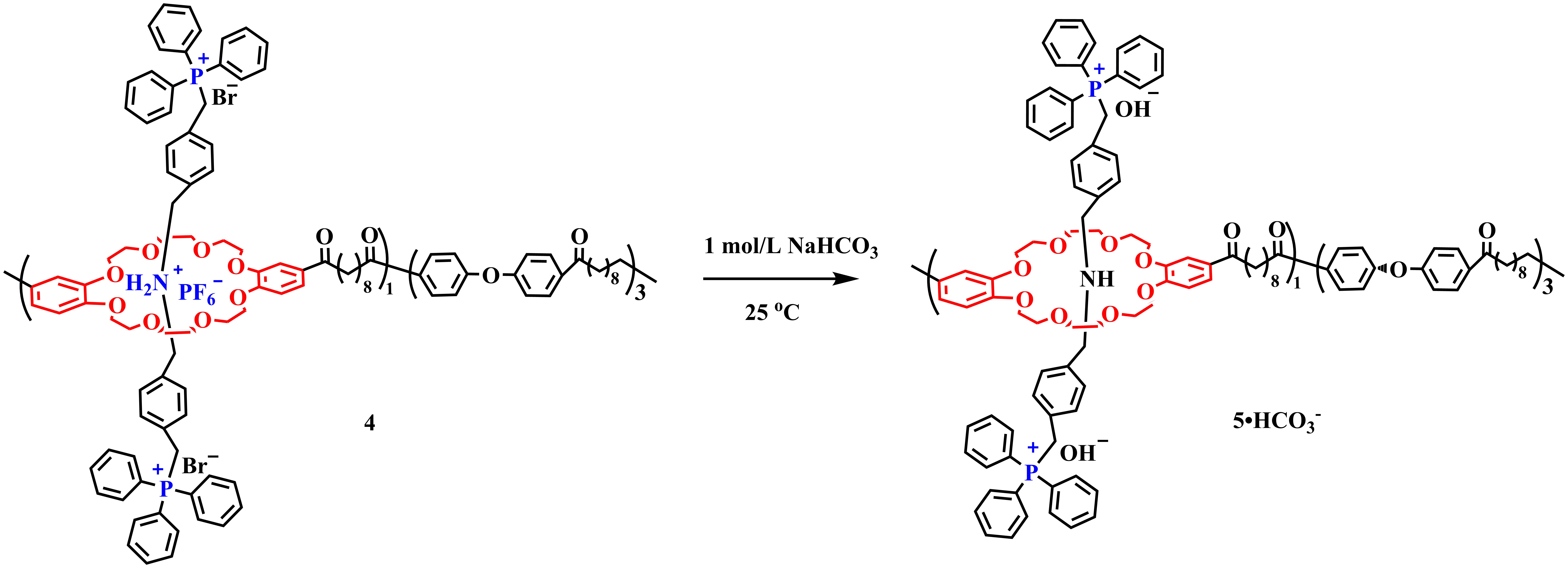


**Scheme S6.** Preparation of polyrotaxane AEMs **5•HCO_3_^-^**


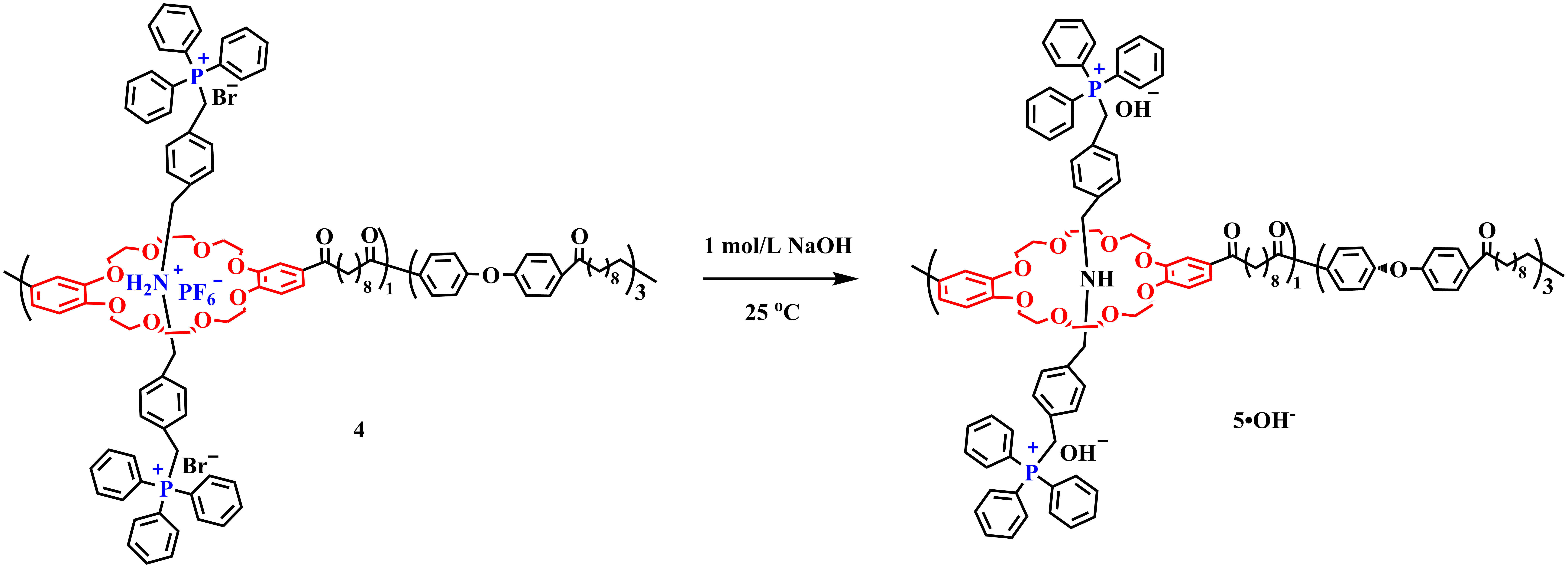


**Scheme S7.** Preparation of polyrotaxane AEMs **5•OH^-^**


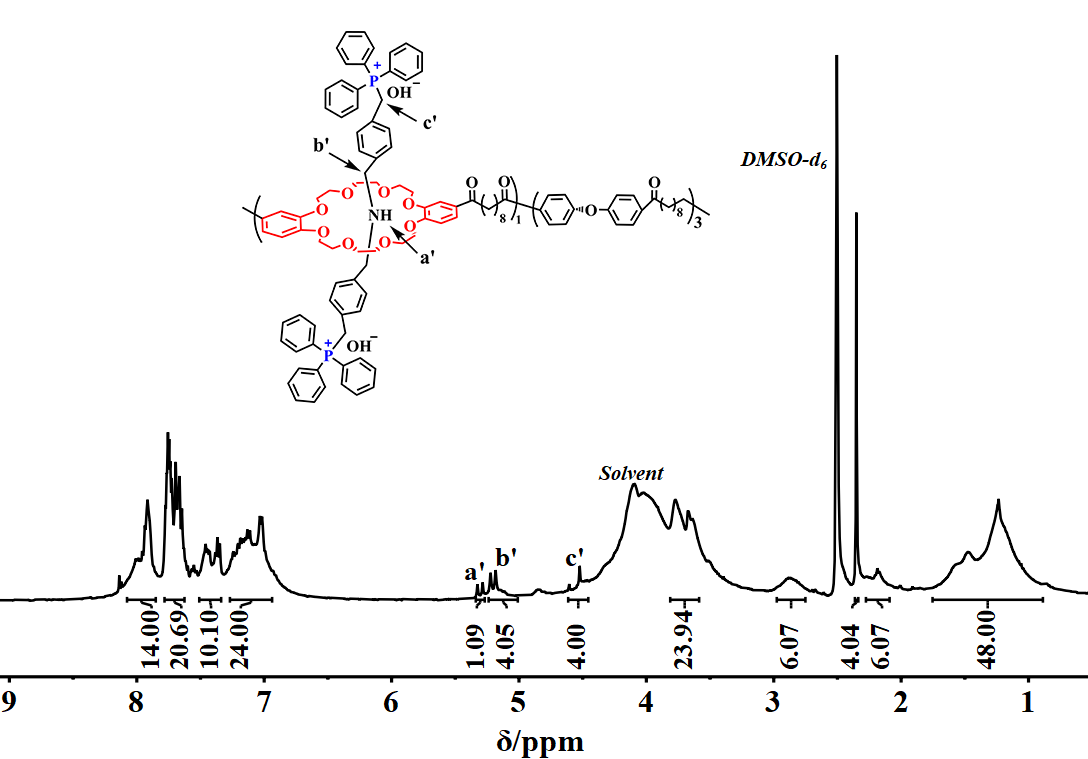


**Figure S8.** ^1^H NMR spectrum of polyrotaxane AEM **5•OH^-^** (400 MHz, DMSO-*d_6_*)

**2.7** **General procedure for rotaxane 6.**

^1^H NMR (400 MHz, DMSO-*d_6_*) δ 7.92 (td, J = 7.4, 1.8 Hz, 6H), 7.73 (td, J = 7.8, 3.5 Hz, 12H), 7.64 (dd, J = 12.6, 7.9 Hz, 12H), 7.41 (s, 2H), 7.10 (d, J = 7.8 Hz, 4H), 6.83 (dd, J = 9.4, 4.6 Hz, 12H), 5.15 – 5.05 (m, 4H), 4.49 (d, J = 8.1 Hz, 4H), 4.03 – 3.92 (m, 8H), 3.62 – 3.52 (m, 8H).


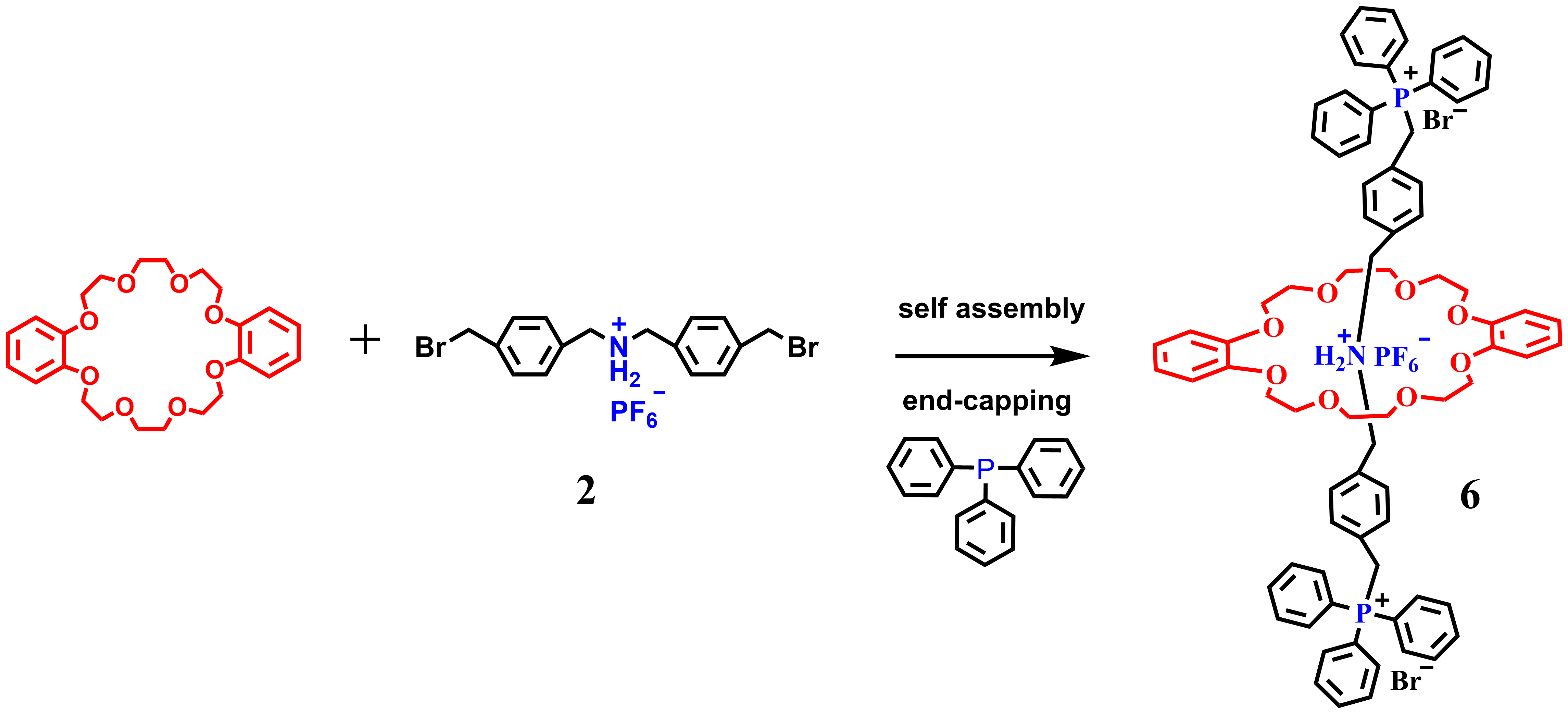


**Scheme S8.** Synthesis of rotaxane **6**


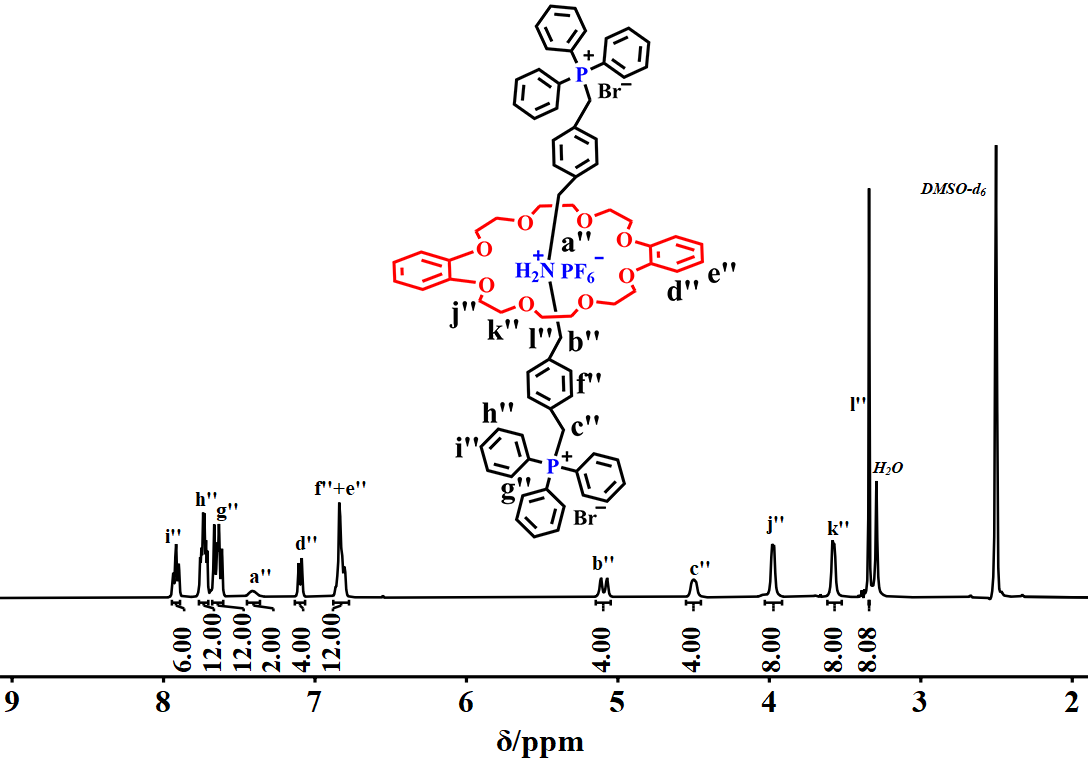


**Figure S9.** ^1^H NMR spectrum of rotaxane **6** (400 MHz, DMSO-*d_6_*)


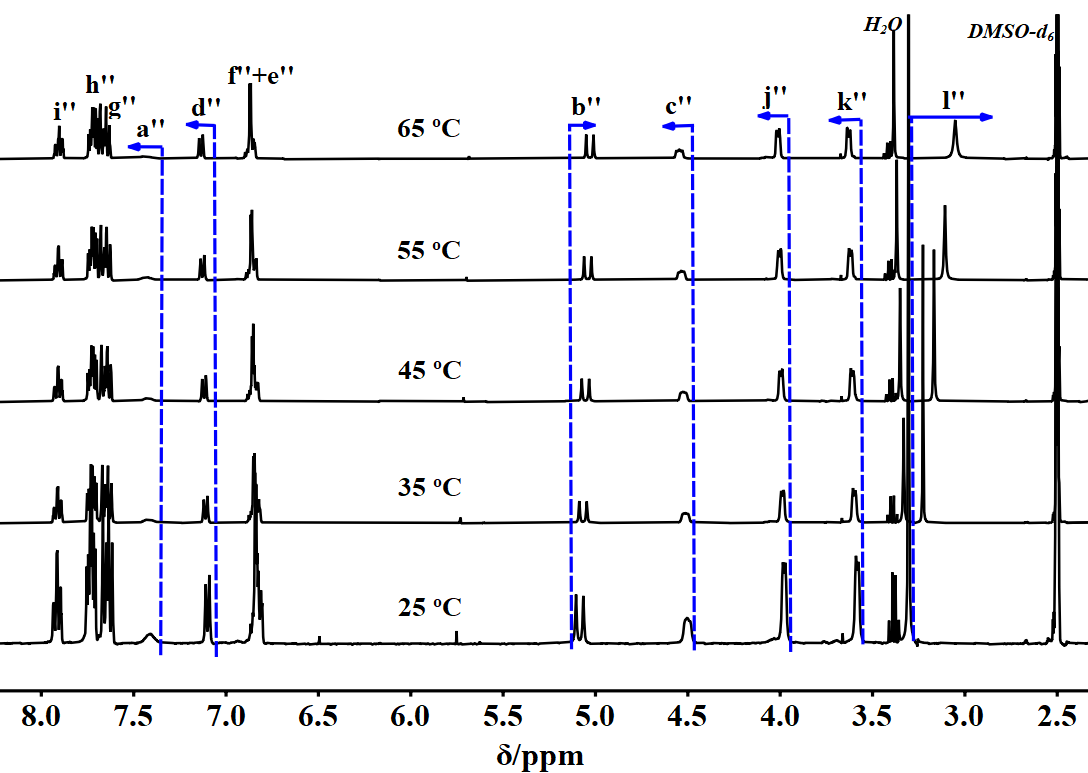


**Figure S10.** Temperature dependent ^1^H NMR spectrum of rotaxane **6** (400 MHz, DMSO-*d_6_*)


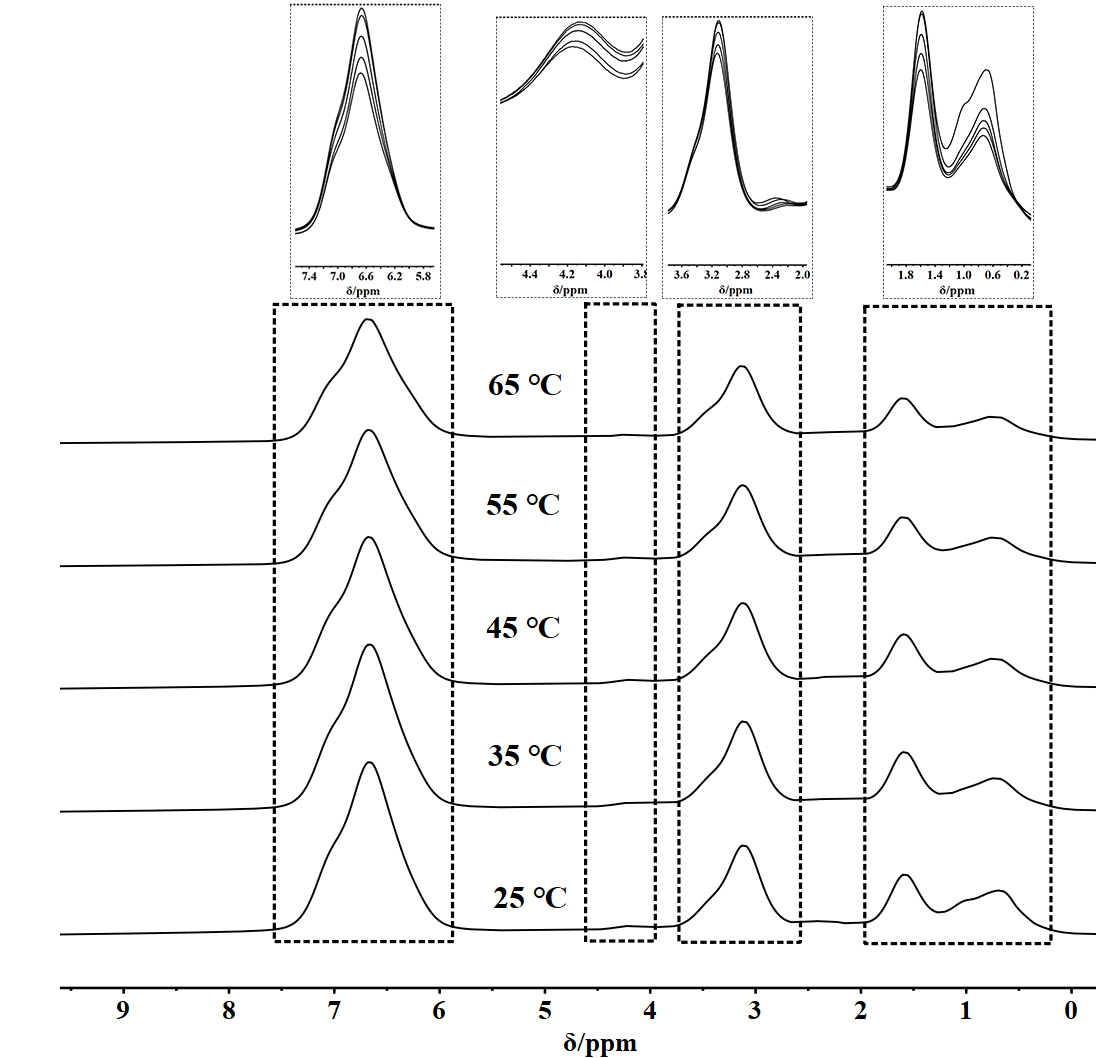


**Figure S11** Temperature-dependent solid-state ^1^H NMR spectra of rotaxane 6 (400 MHz).


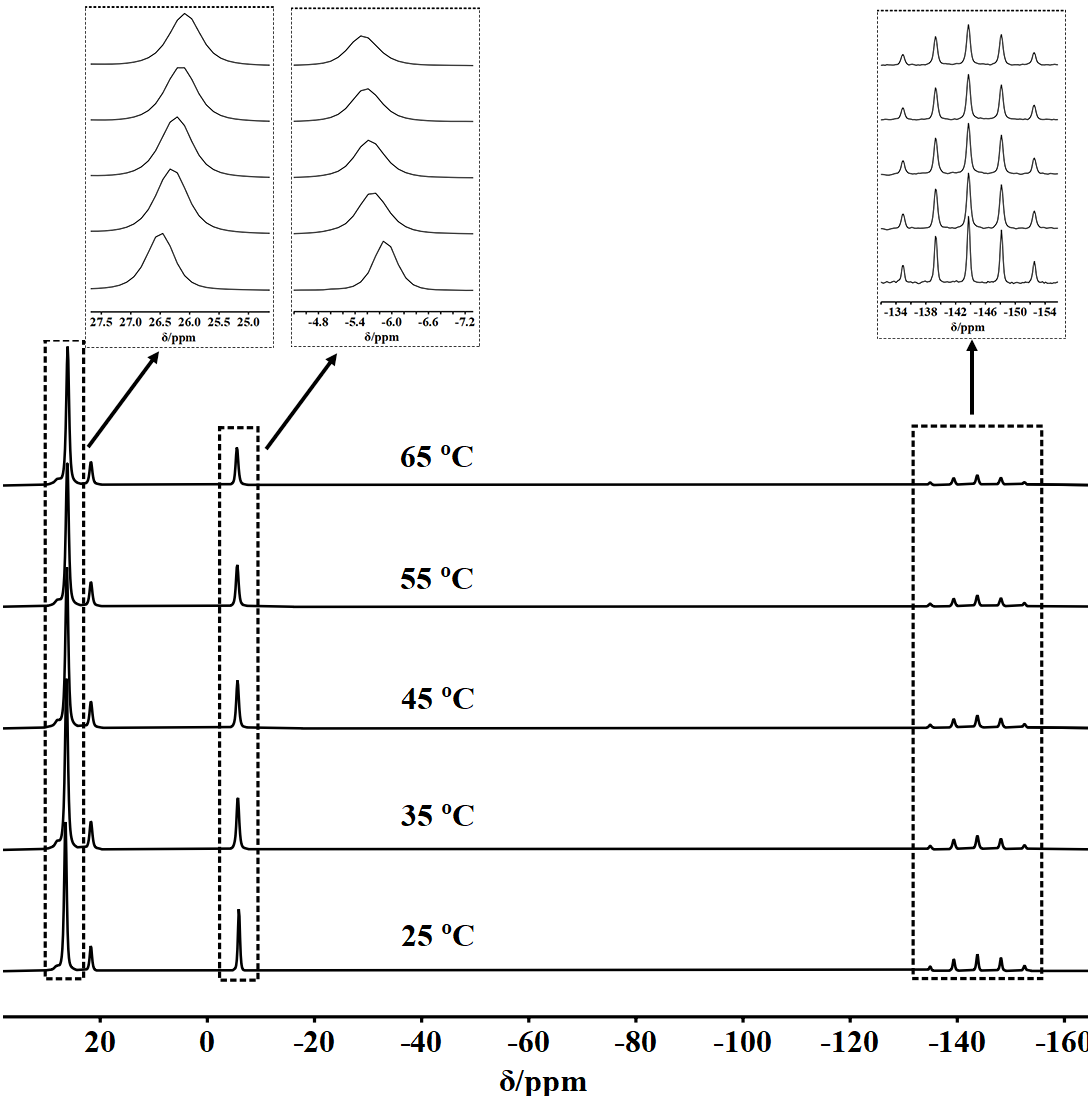


**Figure S12** Temperature-dependent solid-state ^31^P NMR spectra of rotaxane (6) (400 MHz).

**3. COSY 2D NMR of polyrotaxane.**


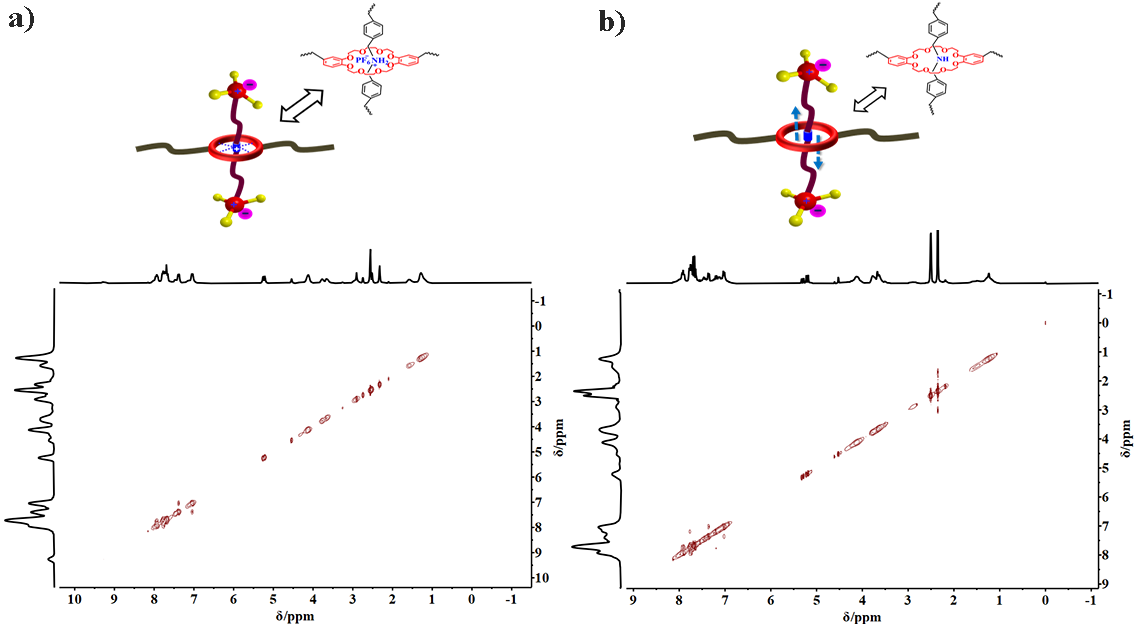


**Figure S13** **a)** COSY 2D NMR of hydrogen-bonded polyrotaxane 4 (400 MHz, DMSO-d6, 298 K); **b)** COSYY 2D NMR of free shuttling polyrotaxane 5 (400 MHz, DMSO-d6,298 K).

**3. Molecular Dynamics Simulations.**

To elucidate the difference of mobility and motion range of cationic groups in polyrotaxane AEM and conventional tethered AEM, an image of angle-distance probability density distribution was drawn according to the coordinates of phosphorus atoms in molecular. In the distribution figure, red area represents high density probability and green area represents low density probability.


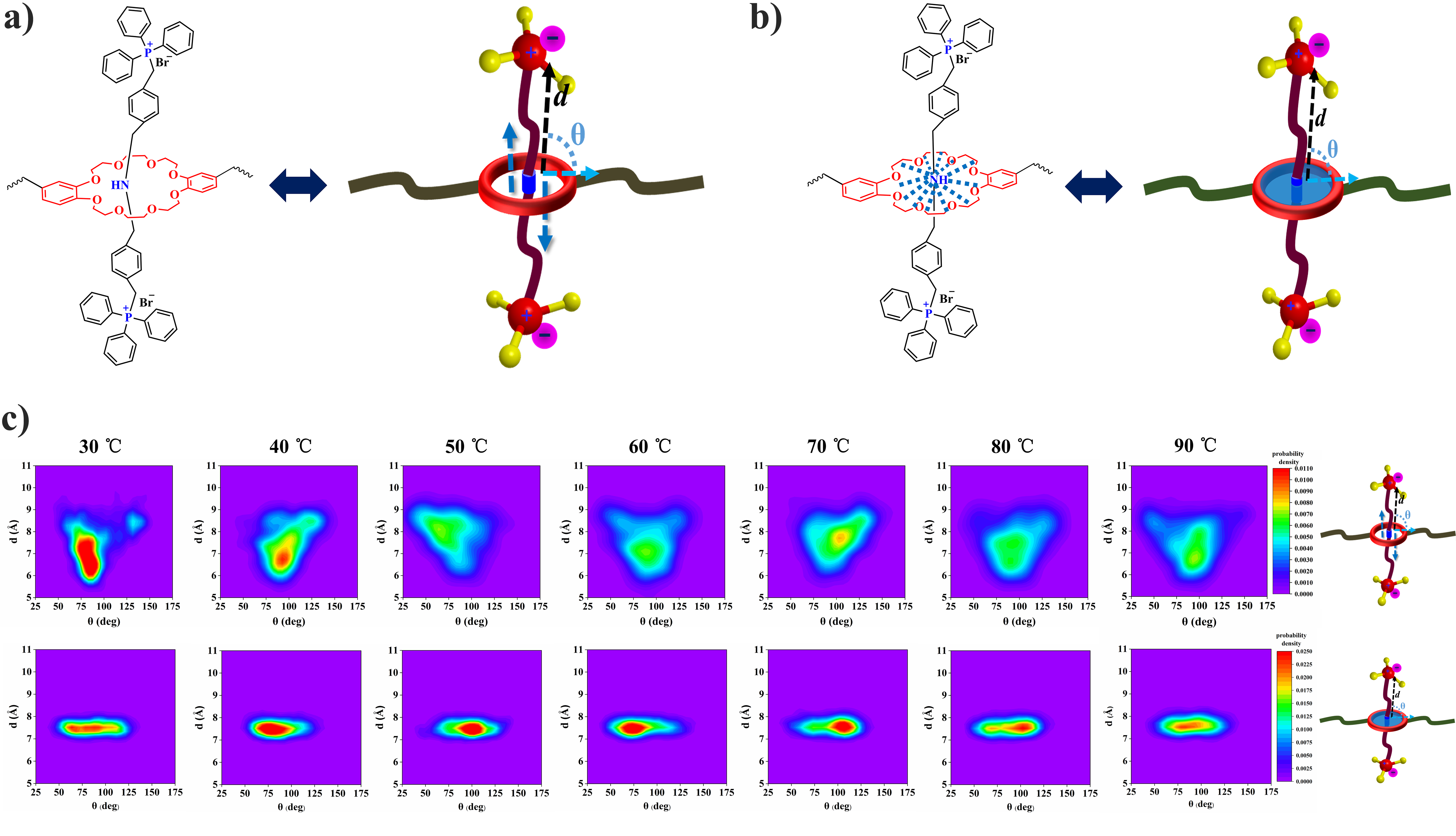


**Figure S14** **a)** Molecular structure of polyrotaxane AEM in molecular dynamics system; **b)** Molecular structure of conventional tethered AEM in molecular dynamics system; **c)** The joint probability density distribution of d and θ for polyrotaxane AEM and conventional tethered AEM.

**4. IEC of polyrotaxane AEMs 4•Br^-^, 5•OH^-^, 5•HCO_3_^-^.**

**Table S1.** IEC values of polyrotaxane AEMs.

| Samples | AEMs 4•Br^-^ | AEMs 5•HCO_3_^-^ | AEMs 5•OH^-^ |
| --- | --- | --- | --- |
| IEC (mmol/g) | 1.08 | 1.17 | 0.85 |

**5. Water uptake (WU) and Dimensional swelling ration (DSR) of polyrotaxane AEMs 4•Br^-^, 5•OH^-^, 5•HCO_3_^-^.**

**Table S2.** WU and DSR of polyrotaxane AEMs

| Sample | WU (%) | | DSR (%) | |
| --- | --- | --- | --- | --- |
|  | 30 ^o^C | 60 ^o^C | 30 ^o^C | 60 ^o^C |
| **AEMs 4•Br^-^** | 62.3 | 205.3 | 8.3 | 35.2 |
| **AEMs 5•HCO_3_^-^** | 85.4 | 243.4 | 10 | 38.5 |
| **AEMs 5•OH^-^** | 41.7 | 261.3 | 5.6 | 41.7 |

**6. Ion conductivity of polyrotaxane AEMs 4•Br^-^, 5•OH^-^, 5•HCO_3_^-^ as a function of temperature.**


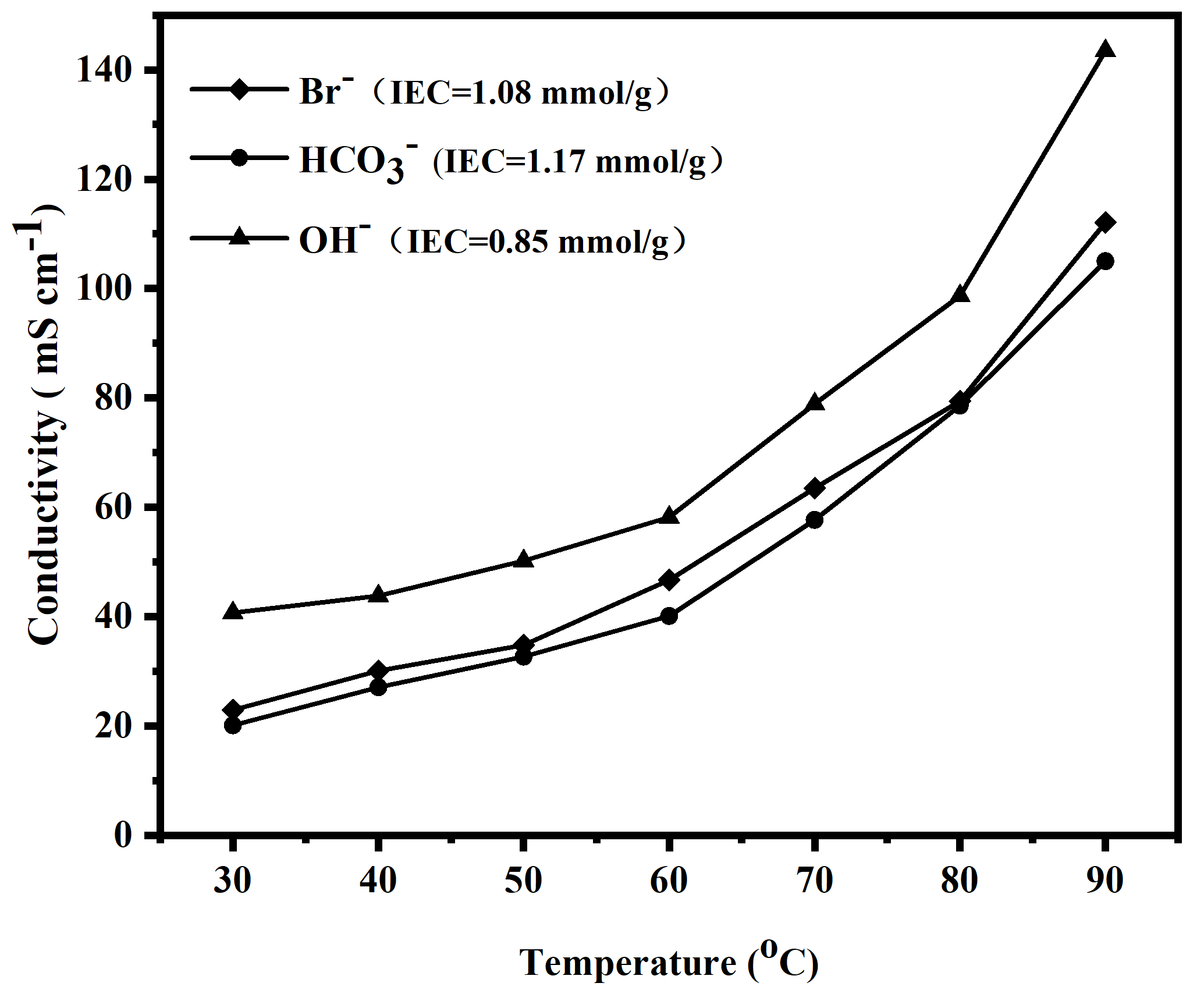


**Figure S15.** Temperature-dependent conductivity of polyrotaxane AEMs

**7. Mechanical strength and thermal stability of polyrotaxane AEMs.**


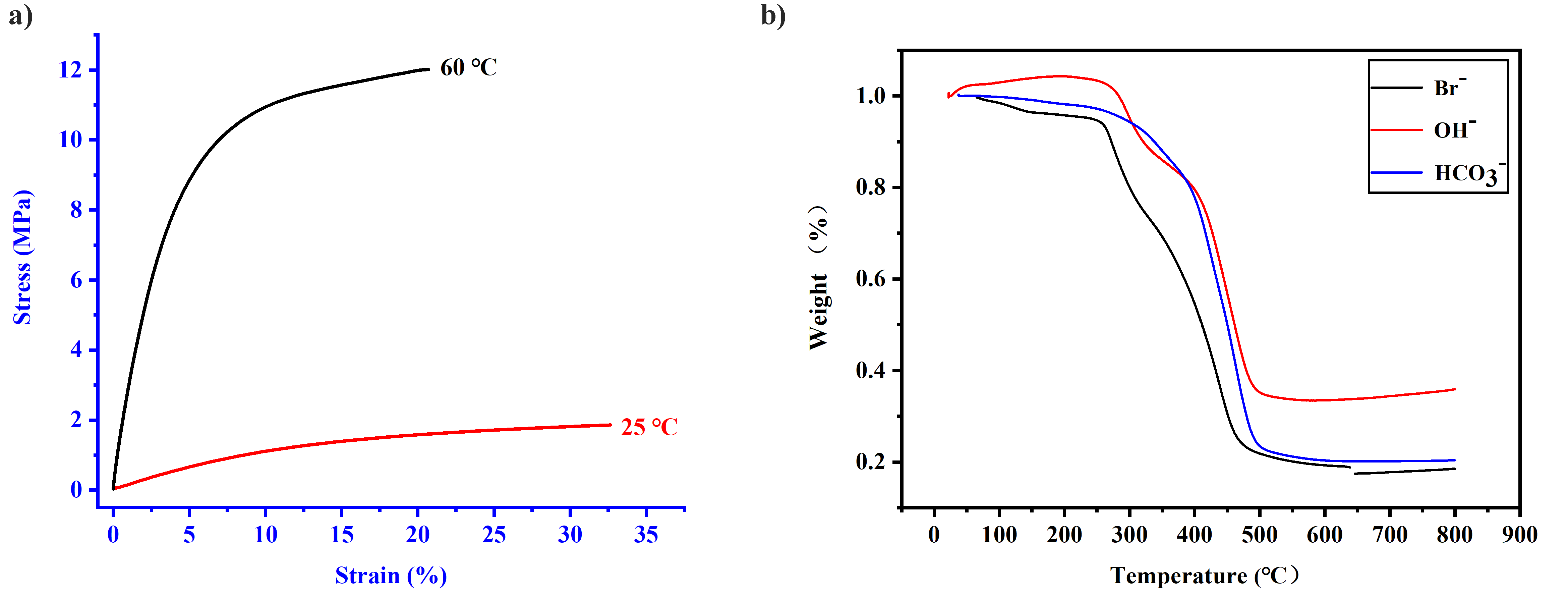


**Figure S16.** **a)** Mechanical properties of the AEMs 4•Br^-^; **b)** TGA curves of the AEMs 4•Br^-^, 5•OH^-^, 5•HCO^3-^.

**8. Schematic illustration of hydroxide ion (OH-) transport via the continuous interconversion between hydration complexes.**


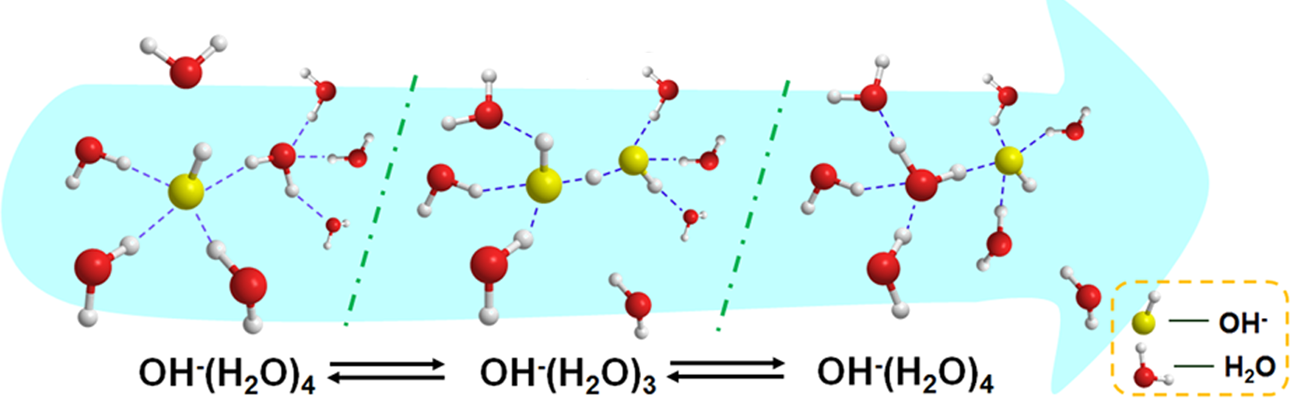


**Figure S17.** Schematic illustration of hydroxide ion (OH-) transport

**9. Change of physical appearance of dry membrane at different temperature.**


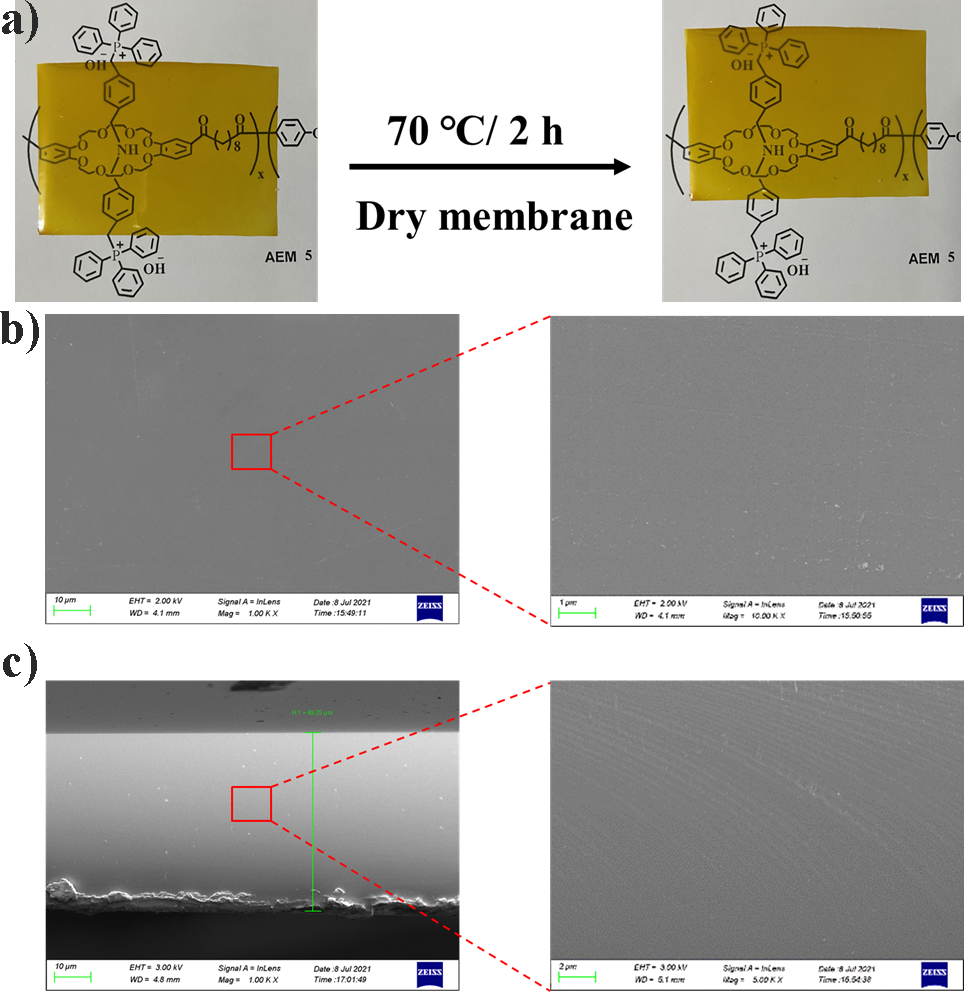


**Figure S18.** **a)** Change of physical appearance of polyrotaxane AEM was heated at 70 ℃ for 2 h; **b)** Surface SEM images of polyrotaxane AEM; **c)** Cross-section SEM images of polyrotaxane AEM.
